# Thymic Epithelial Cell Alterations and Defective Thymopoiesis Lead to Central and Peripheral Tolerance Perturbation in MHCII Deficiency

**DOI:** 10.3389/fimmu.2021.669943

**Published:** 2021-06-15

**Authors:** Francesca Ferrua, Ileana Bortolomai, Elena Fontana, Dario Di Silvestre, Rosita Rigoni, Genni Enza Marcovecchio, Elena Draghici, Francesca Brambilla, Maria Carmina Castiello, Gloria Delfanti, Despina Moshous, Capucine Picard, Tom Taghon, Victoria Bordon, Ansgar S. Schulz, Catharina Schuetz, Silvia Giliani, Annarosa Soresina, Andrew R. Gennery, Sara Signa, Blachy J. Dávila Saldaña, Ottavia M. Delmonte, Luigi D. Notarangelo, Chaim M. Roifman, Pietro Luigi Poliani, Paolo Uva, Pier Luigi Mauri, Anna Villa, Marita Bosticardo

**Affiliations:** ^1^ San Raffaele Telethon Institute for Gene Therapy (SR-Tiget), IRCCS San Raffaele Scientific Institute, Milan, Italy; ^2^ Pediatric Immunohematology and Bone Marrow Transplantation Unit, IRCCS San Raffaele Scientific Institute, Milan, Italy; ^3^ Vita-Salute San Raffaele University, Milan, Italy; ^4^ Human Genome Department, Humanitas Clinical and Research Center, Rozzano, Milan, Italy; ^5^ Milan Unit, Institute of Genetic and Biomedical Research, National Research Council (CNR), Milan, Italy; ^6^ Department of Biomedical Sciences, Institute for Biomedical Technologies-National Research Council (CNR), Milan, Italy; ^7^ Experimental Immunology Unit, Division of Immunology, Transplantation and Infectious Diseases, IRCCS San Raffaele Scientific Institute, Milan, Italy; ^8^ Department of Pediatric Immunology, Hematology and Rheumatology, Necker Children’s Hospital, AP-HP, Paris, France; ^9^ Laboratory “Genome Dynamics in the Immune System”, INSERM UMR1163, Université de Paris, Institut Imagine, Paris, France; ^10^ Centre d’Etude des Déficits Immunitaires, Necker-Enfants Malades Hospital, AP-HP, Paris, France; ^11^ Laboratory of Lymphocyte Activation and Susceptibility to EBV infection, Inserm UMR 1163, University Paris Descartes Sorbonne Paris Cité, Imagine Institute, Paris, France; ^12^ Department of Diagnostic Sciences, Ghent University Hospital, Ghent University, Ghent, Belgium; ^13^ Department of Pediatric Hematology, Oncology and Stem Cell Transplantation, Ghent University Hospital, Ghent, Belgium; ^14^ Department of Pediatrics, University Medical Center Ulm, Ulm, Germany; ^15^ Department of Pediatrics, Medizinische Fakultät Carl Gustav Carus, Technische Universität Dresden, Dresden, Germany; ^16^ Cytogenetics and Medical Genetics Unit and “A. Nocivelli” Institute for Molecular Medicine, Spedali Civili Hospital, Department of Molecular and Translational Medicine, University of Brescia, Brescia, Italy; ^17^ Unit of Pediatric Immunology, Pediatrics Clinic, University of Brescia, ASST-Spedali Civili Brescia, Brescia, Italy; ^18^ Translational and Clinical Research Institute, Newcastle University, Newcastle upon Tyne, United Kingdom; ^19^ Department of Pediatric Immunology and HSCT, Great North Children's Hospital, Newcastle upon Tyne, United Kingdom; ^20^ Autoinflammatory Diseases and Immunodeficiencies Center, IRCCS Istituto G. Gaslini, and Department of Neurosciences, Rehabilitation, Ophthalmology, Genetics, and Maternal and Children's Sciences, University of Genoa, Genoa, Italy; ^21^ Division of Blood and Marrow Transplantation, Children's National Hospital, Washington, DC, United States; ^22^ Laboratory of Clinical Immunology and Microbiology, NIAID, NIH, Bethesda, MD, United States; ^23^ Division of Immunology & Allergy, Department of Pediatrics, The Hospital for Sick Children, the Canadian Centre for Primary Immunodeficiency and the University of Toronto, Toronto, ON, Canada; ^24^ Department of Molecular and Translational Medicine, Pathology Unit, University of Brescia, Brescia, Italy; ^25^ CRS4, Science and Technology Park Polaris, Pula, Cagliari, Italy

**Keywords:** thymus, thymic epithelial cells, primary immunodeficiency, MHCII, central tolerance

## Abstract

Major Histocompatibility Complex (MHC) class II (MHCII) deficiency (MHCII-D), also known as Bare Lymphocyte Syndrome (BLS), is a rare combined immunodeficiency due to mutations in genes regulating expression of MHCII molecules. MHCII deficiency results in impaired cellular and humoral immune responses, leading to severe infections and autoimmunity. Abnormal cross-talk with developing T cells due to the absence of MHCII expression likely leads to defects in thymic epithelial cells (TEC). However, the contribution of TEC alterations to the pathogenesis of this primary immunodeficiency has not been well characterized to date, in particular in regard to immune dysregulation. To this aim, we have performed an in-depth cellular and molecular characterization of TEC in this disease. We observed an overall perturbation of thymic structure and function in both MHCII^−/−^ mice and patients. Transcriptomic and proteomic profiling of murine TEC revealed several alterations. In particular, we demonstrated that impairment of lymphostromal cross-talk in the thymus of MHCII^−/−^ mice affects mTEC maturation and promiscuous gene expression and causes defects of central tolerance. Furthermore, we observed peripheral tolerance impairment, likely due to defective Treg cell generation and/or function and B cell tolerance breakdown. Overall, our findings reveal disease-specific TEC defects resulting in perturbation of central tolerance and limiting the potential benefits of hematopoietic stem cell transplantation in MHCII deficiency.

## Introduction

Major Histocompatibility Complex (MHC) class II (MHCII) deficiency (MHCII-D), also known as Bare Lymphocyte Syndrome (BLS), is a rare autosomal recessive combined immunodeficiency (CID) due to mutations in genes regulating expression of MHCII molecules [OMIM#209920] (*CIITA*, *RFXANK, RFX5*, *RFXAP*) (1–4). MHCII molecules play a crucial role in immune responses, due to their fundamental role in presenting exogenous peptides to the antigen T-cell receptor (TCR) of CD4^+^ T lymphocytes. In the thymus, complexes formed by self-peptides and MHCII, expressed on thymic epithelial cells (TEC) and dendritic cells, drive the positive and negative selection processes, which are critical for the development of CD4^+^ T cells and the establishment of a non-self-reactive TCR repertoire (1, 4). In the periphery, MHCII molecules contribute to the induction of antigen-specific immune responses against pathogens and tumors and, through the presentation of self-peptides, to the maintenance of peripheral T-cell tolerance.

Defects of antigen processing and presentation via MHCII molecules in patients with MHCII deficiency lead to impairment of thymic education and peripheral T-cell help (5, 6). Thymopoiesis is defective in this disease, presumably due to impaired thymic selection in the absence of MHCII on TEC (4, 7). Examination of thymic biopsies from two patients with CID and defective expression of MHC I and II molecules revealed normal lobular thymic architecture, with distinct cortex-medullary areas, well-differentiated epithelium, and presence of Hassall’s corpuscles. However, MHCII antigen expression was not detectable on epithelial cells in the cortex and absent/reduced in the medulla, in contrast to what was observed in normal controls (8). Study of thymic function in 8 MHCII-deficient patients (7) showed that, despite normal TCR-Vβ repertoire of total CD3^+^ T cells, clonal abnormalities emerged at flow cytometric evaluation of TCR-Vβ repertoire on CD4^+^ T cells and at spectratyping evaluation of TCR-Vγ repertoire on total CD3^+^ lymphocytes (7). These findings suggest a reduced global thymic activity in MHCII deficiency and emphasize the key role of MHCII molecules in the process of normal thymic maturation of T lymphocytes. However, interestingly, TCR excision circles (TREC) were detectable in patients’ total lymphocytes and sorted CD4^+^ cells, reflecting normal early T-cell development (4, 7).

In MHCII-D patients, lack of MHCII molecules expression on the cell surface results in severe CD4^+^ T-cell lymphopenia and impaired antigen-specific cellular and humoral immune responses. Usually hematopoiesis is not affected, and most patients have normal numbers of circulating B and total T cells. Despite intact response to mitogen stimulation, T-cell mediated responses to foreign antigens are impaired. Hypogammaglobulinemia is a common finding (1, 4) and antibody responses to immunizations and to infections by microbial agents are generally absent or markedly reduced (6). On the other hand, autoantibodies have been demonstrated in several patients (6) and autoimmune manifestations, such as autoimmune cytopenias, have been observed in 6–20% of patients (4, 9, 10). Patients suffer from recurrent and severe infections (viral, bacterial, fungal, and protozoan), primarily involving the respiratory and gastrointestinal tract (4, 11) since early in life. Inflammatory enteropathy is frequently observed and reflects the multifaceted disturbance of intestinal epithelial cell regulation of adaptive mucosal immunity due to the lack of MHCII expression on enterocytes (12). If untreated, MHCII deficiency is often fatal early in life (3, 4). Allogeneic hematopoietic stem cell transplantation (HSCT), preferably from an HLA-identical sibling donor, is the treatment of choice, but overall success rate is limited (10, 13–15). In recent years HSCT survival has improved, but CD4^+^ T-cell lymphopenia may persist (16).

The Aβ^0/0^ mouse model, lacking MHC class II antigens, shares some phenotypical features with MHCII-D (17, 18). Aβ^0/0^ mice have barely detectable numbers of CD4^+^ T lymphocytes in secondary lymphoid organs, while in the thymus, immature CD4 single-positive (SP) thymocytes are present, indicating impairment of positive selection process, particularly in its initial stages, when TCR/MHC interactions are required (19). Interestingly, it has been shown that a large proportion of residual CD4^+^ T cells correspond to CD1-restricted natural killer T (NKT) cells in MHCII-deficient mice (20, 21). *In vivo* treatment of Aβ^0/0^ mice with anti-TCR antibody has been shown to restore the generation of circulating CD4^+^ T cells and to normalize the thymic medulla (22). A reduction of the medullary TEC (mTEC) compartment has been described also in another MHCII ko mouse model (Aα^−/−^ mice) (23). Reduced number of mature mTECs and decreased expression of Aire and Aire-dependent and -independent tissue restricted antigens (TRA) have been detected in the thymus of Aα^−/−^ mice (24). The demonstration of CD8^+^ T cell infiltrates in multiple organs of Aα^−/−^ mice suggested defects of central tolerance and/or of regulatory T (Treg) cells (24). While CD4^+^ FoxP3^+^ Treg cells were not found in the thymus of Aα^−/−^ mice, they were present in the periphery and seemed functional and efficient in mediating immune suppression (25). Furthermore, in experimentally induced colitis models, regulatory CD25^+^ double-positive (DP) T cells generated in MHCII ko mice (Aα^−/−^ or Aβ^0/0^), probably arising from SP CD8^+^ T cells, have been demonstrated to control the colitogenic potential of CD25^−^CD4^+^ T cells (26). Indeed, CD8^+^ T cells constitutively expressing CD25 and bearing characteristics similar to regulatory CD4^+^CD25^+^ T cells have been also detected in the thymus of MHCII^−/−^ mice (27).

In conclusion, it is currently unclear if TEC defects are responsible, at least in part, for the pathogenesis of MHCII deficiency. To better define this issue, here we report on thymic defects in both patients and in the Aβ^0/0^ mouse model of MHCII-D and describe how these alterations lead to peripheral immune dysregulation.

## Materials and Methods

### Human Samples

A thymic biopsy was obtained from a 23-month-old infant with MHCII deficiency upon informed consent in accordance with the Research Ethics Board at The Hospital for Sick Children in Toronto (Canada). Patient’s data were compiled prospectively and retrospectively from medical records and entered into the Primary Immunodeficiency Registry and Tissue Bank (REB protocol no. 1000005598). The patient presented at 18 months of life with a history of recurrent respiratory tract infections, chronic diarrhea, and CMV hepatitis. She had a family history of MHCII deficiency and was found to be homozygous for a mutation in the *CIITA* gene. Immunological data are reported in **Table 1**. The patient received a matched related HSCT, but engraftment was poor.

**Table 1:** Immunological work-up of the MHCII-D patient who underwent thymic biopsy.

Test	Results
Proliferative response to mitogens	
–PHA	40–50% of control
–Response to specific Ag at day 6(PPD, VZV, CMV, HSV, Candida)	No
Serum immunoglobulins	Normal
Immune phenotype	
CD3	64% (3,184/μl)
CD4	7.1% (356/μl)
CD8	44% (2,241/μl)
CD56	3% (151/μl)
CD19	34% (1,713/μl)

Abnormal results according to age-based reference ranges (28) are reported in bold.

PHA, phytohemagglutinin; Ag, antigen; PPD, tuberculin-purified protein derivative; VZV, Varicella-Zoster virus; CMV, Cytomegalovirus; HSV, Herpes Simplex virus.

A human thymic sample from a healthy control used for comparison was analyzed retrospectively in compliance with Declaration of Helsinki and policies approved by Ethics Board of Spedali Civili in Brescia. Specifically, for retrospective and exclusively observational study on archival material obtained for diagnostic purposes, patient consent was not needed (Delibera del Garante n. 52 del 24/7/2008 and DL 193/2003).

Blood samples were collected from 11 patients with MHCII deficiency [peripheral mononuclear cells (PBMC), n = 4; serum or plasma, n = 9]. Each patient was attributed a numerical code (MHCII_xx). Questionnaires to gather patient’s relevant clinical data were sent to referring clinicians, who were responsible for the collection of informed consent for biological samples’ collection and anonymized biological sample/data sharing from their own patients, according to local research protocols, reviewed and approved by local ethics committees or institutional review board (IRB) [for NIH patient’s samples, protocols 18-I-0041 and 18-I-0128, approved by the NIH IRB]. Data about clinical history, immunological features and HSCT of this cohort of MHCII-D patients are reported in **Tables 2**–**4** respectively.

**Table 2:** MHCII-D patients’ features.

Patient code	Mutated gene	Age at sampling (years)	Pre/Post HSCT	Infections	Immune dysregulation	Chronic diarrhea
MHCII_01	NK	0.5	Pre	Yes	No	Yes
MHCII_02	RFXANK	12.8	Pre	Yes	AIHA	Yes
MHCII_03	RFXANK	0.9	Pre	Yes	No	No
MHCII_04	RFXANK	4.0	Pre	Yes	No	Yes
MHCII_05	RFXAP	16.0	Pre	Yes	Autoimmune enteropathy and polyendocrinopathy^#^	Yes
MHCII_08	RFXANK	6.5	Post (+2 years)	Yes	No	Yes
MHCII_09*	RFXANK	4.7	Pre	Yes	No	Yes
MHCII_10*	RFXANK	4.6	Pre	Yes	No	Yes
MHCII_11	NK	0.4	Pre	Yes	No	NK
MHCII_12	RFXANK	24.1	Pre	Yes	No	Yes
MHCII_13	RFXANK	15.5	Pre	Yes	AIHA, autoimmune thyroiditis, adrenal insufficiency	No

NK, not known; HSCT, hematopoietic stem cell transplantation; AIHA, autoimmune hemolytic anemia; mo., months; Ab, antibody. ^#^Insulin-dependent diabetes mellitus (IDDM) type 1, hypothyroidism.

*These patients are described in greater detail in (16).

**Table 3:** Immunophenotype and serum Ig level in our cohort of MHCII-D patients.

Patient code	CD3^+^ cells (×10^9^/l)	CD4^+^ cells (×10^9^/l)	CD8^+^ cells (×10^9^/l)	CD19^+^ cells (×10^9^/l)	CD16^+^/CD56^+^ cells (×10^9^/l)	HLA-DR^+^ cells (×10^9^/l)	IgM (g/L)	IgG (g/L)	IgA (g/L)
MHCII_01	1,495	402	1,087	491	30	Absent	0.06	4.94	<0.02
MHCII_02	1,121	240	741	261	20	Absent	0.31	9.93	<0.05
MHCII_03	1,021	484	480	2,178	77	7	0.29	3.57	0.2
MHCII_04	NA	NA	NA	NA	NA	NA	NA	NA	NA
MHCII_05	NA	NA	NA	NA	NA	NA	NA	NA	NA
MHCII_08	1,540	901	526	1,315	714	Present^§^	1.17	9.26	<0.05
MHCII_09*	207	71	117	58	34	Absent	0.27	4.1°	0.0
MHCII_10*	1,247	535	563	243	<16	Absent	0.21	18.2	0.07
MHCII_11	NA	NA	NA	NA	NA	NA	NA	NA	NA
MHCII_12	725	136	444	136	45	11 (tot. ly.), 1 (B cells)	0.004	12.2	0.006
MHCII_13	1,947	331	1,512	62	21	41	<0.13	8.07	<0.07

Data are reported at blood sampling. Abnormal results according to age-based reference ranges (28) are reported in bold.

°Not on Ig replacement therapy at sampling. ^§^Patient with 100% donor chimerism after HSCT. ND, not done; NK, not known; NA, not available; ly., lymphocytes.

*These patients are described in greater detail in (16).

**Table 4:** MHCII-D patients’ treatment features.

Patient code	HSCT	Age at HSCT (years)	Donor	HSC source	Conditioning regimen	Complications after HSCT	Donor chimerism^§^	HSCT outcome^§^
MHCII_01	Yes°	1.5	MUD	PBSC	Bu/Flu/ ATG	GVHD, respiratory insufficiency	NK	Deceased (+1.8 mo. after HSCT)
MHCII_02	No	–	–	–	–	–	–	–
MHCII_03	Yes°	1.1	MUD	BM	Bu/Cy/ ATG	No	31% T cells, 8% B cells	Alive and well
MHCII_04	Yes°	4.0	MUD	BM	Treo/Flu/TT/ Alemtuzumab	Mucositis, CLS, brain hemorrhage	NK	Deceased (+25 days after HSCT)
MHCII_05	Yes°	16.2	MUD	BM	Bu/Flu/TT/ Alemtuzumab	VOD, GVHD, infections, cytopenias	100%	Deceased (+5 mo. after HSCT)
MHCII_08	Yes	4.5	MRD	BM	Bu/Flu/ ATG	GVHD, TMA	100%	Alive and well (but lung sequelae)
MHCII_09*	Yes°	5.2	MUD	PBSC	Treo/Flu/ Alemtuzumab	Viral infections	100%	Alive and well
MHCII_10*	Yes°	6.1	TCRαβ/CD19- depleted Haplo	PBSC	Treo/Flu/TT/ ATG/Rtx	GVHD, severe infections, BM failure	100% (after T-cell add-back + top up haplo tx)	Deceased (+6.5 mo. after first HSCT)
MHCII_11	No	–	–	–	–	–	–	Deceased
MHCII_12	No	–	–	–	–	–	–	–
MHCII_13	No	–	–	–	–	–	–	–

ATG, anti-thymocyte globulin; BM, bone marrow; Bu, Busulfan; CLS, capillary leak syndrome; Cy, cyclophosphamide; Flu, fludarabine; GVHD, graft-versus-host disease; HSC, hematopoietic stem cell; mo., months; MRD, matched related donor; MUD, matched unrelated donor; NK, not known; PBSC, peripheral blood stem cells; Rtx, rituximab; TMA, thrombotic microangiopathy; Treo, Treosulfan; TT, thiotepa; VOD, veno-occlusive disease.

°performed after sample sampling. ^§^at last available follow up.

*These patients are described in greater detail in (16).

Peripheral blood (PB) from healthy controls (HD) was obtained in accordance with the 1964 Declaration of Helsinki and its subsequent amendments, under biological material collection protocols approved by the Institutional Ethical Committee of San Raffaele Hospital (Tiget07, Tiget09). Informed consent was signed directly by the subject or by parents or legal representatives in case of minors.

### Mice

Aβ^0/0^ mice were kindly provided by Dr. Matteo Iannacone (San Raffaele Scientific Institute, Milan, Italy). *Rag1* ^−/−^ mice were purchased from The Jackson Laboratories. C57/black (BL) 6 control wild-type (WT) mice were purchased from Charles River Laboratories Inc. All mice were housed in specific pathogen-free conditions and treated according to protocols approved by the Animal Care and Use Committee of the San Raffaele Scientific Institute (Institutional Animal Care and Use Committee protocol no. 710 and 712).

### Histological Analysis

Following sacrifice, thymus and gut tissue isolated from mice were formalin-fixed and paraffin-embedded. Hematoxylin and Eosin (H&E) staining was used to assay basic histopathological changes. Paraffin sections were de-waxed, rehydrated, endogenous peroxidase activity was blocked by 0.1% H_2_O_2_, and nonspecific background reduced with Rodent Block M (Biocare Medical, Concord, CA, USA) before heat-based antigen-retrieval treatment and incubation with antibodies. Depending on the primary antibodies used, sections were incubated with Rat-on-Mouse HRP-Polymer (Biocare Medical) or MACH 1™ Universal HRP Polymer Kit (Biocare Medical) or 4plus Streptavidin HRP label (Biocare Medical); reactions were developed in Biocare's Betazoid DAB and nuclei counterstained with Hematoxylin. Digital images were acquired with an Olympus XC50 camera mounted on a BX51 microscope (Olympus, Tokyo, Japan), with CellF Imaging software (Soft Imaging System GmbH, Münster, Germany) or with a Nikon Eclipse E600 Microscope (Nikon) with NIS Elements Software (Nikon). The following primary antibodies were used on thymic sections: rabbit anti-cytokeratin 5 (CK5) (1:200; Covance), rat anti-cytokeratin 8 (CK8) (1:200; Developmental Studies Hybridoma Bank); rat anti-mouse AIRE (1:300; Millipore); rat anti-mouse FOXP3 (1:100; eBioscience); biotin-UEA (Biotinylated Ulex Europaeus Agglutinin I) (1:800; Vector Laboratories). Rabbit anti-CD3 primary antibody (1:100; ThermoFisher Scientific) was used on gut sections.

To quantify abnormalities of colon pathology, a histological score was used. The degree of colic alterations was blindly graded using combined scores including: the grade of inflammation (grade 0, no evidence of inflammation; grade 1, low; grade 2, moderate; grade 3, high; grade 4, intense and diffuse level of inflammation), the structural changes of intestinal layers (grade 0, absence; grade 1, focal; grade 2, partial; grade 3, diffuse level of structural changes), and the gland secretion alterations (grade 0, normal; grade 1, slight gland secretion alterations; grade 2, moderate; grade 3, diffuse gland secretion alterations). The cumulative total scores ranged from 0 to 10.

Human thymic tissue sample was formalin-fixed and paraffin-embedded. Sections were used for routine H&E staining and treated as described above. Nonspecific background was reduced with Background Sniper (Biocare Medical, Concord, CA, USA) before heat-based antigen-retrieval treatment and incubated with primary antibodies. The following primary antibodies were used: rat anti-human FOXP3 (1:100; eBioscience), mouse anti-human AIRE (1:3000; kindly provided by Prof P. Peterson, University of Tartu, Tartu, Estonia), biotin-UEA (Biotinylated Ulex Europaeus Agglutinin I) (1:800; Vector Laboratories) and Claudin-4 (used according to local standards of Pathological Anatomy Unit of Spedali Civili, Brescia, Italy). Sections were processed as described above for the murine experiments. Morphometric analysis was performed using Olympus Slide Scanner VS120-L100 (Olympus, Tokyo, Japan) and Image-pro software (Olympus) was used to analyze them. Digital images were acquired with the same instruments and software as described above.

### Murine Cell Isolation 

Murine TEC isolation was performed on age-matched WT and Aβ^0/0^ mice. Briefly, mice were sacrificed by decapitation to avoid excessive bleeding during surgery. Murine thymus was cleaned from fat and stromal tissues, and then digested at 37°C with an enzymatic solution containing Liberase TL and DNAse I (Roche). Digested tissues were collected in DMEM (Lonza) supplemented with 10% fetal bovine serum (FBS), 1% glutamine and 1% penicillin and streptomycin. Single thymic cell suspensions were then incubated with anti-CD45 micro-beads (Miltenyi Biotec) and processed with the AutoMACS Pro Separator (Miltenyi Biotec). The CD45 negative fraction was retrieved and then tested by multicolor FACS analyses for the expression of TEC markers.

Thymocytes, splenocytes and lymphocytes were freshly isolated by mechanically disrupting the thymus, the spleen and lymph nodes of age-matched WT and Aβ^0/0^ mice. In addition, splenocytes were lysed with Red Cell Lysis Buffer (RCLB) (150mM M NH4Cl, 10 mM KHCO3, and 0.1 mM EDTA). PB samples underwent red blood cell (RBC) lysis twice with RCLB, before further processing. Recovered cells were re-suspended in D-PBS (EuroClone, Pero, Italy) with 2% FBS and subsequently stained for flow cytometric analysis. CD4^+^ T cells were isolated by pooled spleens and mesenteric lymph nodes (MLNs) from 2–3 mice/pool of age-matched WT and Aβ^0/0^ mice using CD4-specific magnetic beads via negative selection according to the manufacturer’s instructions (CD4^+^ isolation kit Miltenyi Biotec).

### Flow Cytometric Analysis of Murine Cells

The list of monoclonal antibodies used for the staining of murine TEC, thymocytes, splenocytes and lymphocytes derived from lymph nodes are reported in the *Materials and Methods* section in this article’s **Supplementary Materials**. Cells were acquired on a FACS CANTO (BD Pharmingen) and analyzed with FlowJo software.

### Cell Sorting of Murine TEC

TECs were isolated and enriched with the AutoMACS Pro Separator after digestion of thymi from pool of 5–10 age-matched WT and Aβ^0/0^ mice of 4–6 weeks of age, as previously described. To sort mTEC and cTEC, isolated TECs were stained with anti-CD45 (30-F11), anti-CD326 (EpCam; G8.8), anti-MHCII (M5 114.15.2) (all Biolegend), anti-Ulex-1 (FL 1061, Vector) and anti-Ly51 (6C3, Miltenyi) monoclonal antibodies and sorted with the MoFlo Legacy cell sorter (Becton Dickinson), with 100 μl noozle (FRACTAL facility of San Raffaele Hospital, Milan, Italy). Non-viable cells were excluded from analyses using 7-AAD (BD Pharmingen). In order to preserve RNA quality for further analyses, ProtectRNA^TM^ RNase Inhibitor (Sigma-Aldrich) was added to CD45-negative fractions according to manufacturer’s instruction, before sorting procedure. Moreover, TEC subsets were sorted directly in RNAlater (Sigma-Aldrich), kept at +4°C overnight and then at −20°C until use.

### RT-PCR

RNA extraction from sorted TEC from WT and Aβ^0/0^ mice was performed with RNeasy microkit (QIAGEN, Hilden, Germany) according to manufacturer’s instructions. RNA was then stored at −80°C until use. Reverse transcription of murine-sorted TEC mRNA, synthesis and amplification of cDNA were performed with the Ovation PicoSL WTA System V2 (Nugen), according to the manufacturer’s instructions. Purification of amplified cDNA was achieved using the QIAquick PCR Purification Kit (QIAGEN). Quantitative Real-time (RT) PCR was performed using Fast SYBR green master Mix (Thermo Fisher Scientific) and the ViiA7 Real-Time PCR System (Thermo Fisher Scientific). Each sample was analyzed in duplicate. The relative level of expression was determined by normalization to β-actin (Actb) ribosomal RNA. The primers used are listed in **Supplementary Table 1**.

### Bulk RNA-Seq

The cDNA libraries of sorted cTEC and mTEC from WT and Aβ^0/0^ mice were constructed using the Takara SMART-Seq v4 Ultra low input RNA Kit. PCR library products were quantified using the Agilent DNA 1000 assay (Agilent#5067-1504) on an Agilent Technologies 2100 bioanalyzer. Pooled libraries were loaded on a Paired End Flow Cell using the cBot System (Illumina) and the HiSeq 3000 platform. At the end of the run, around 30 M of 50 bp paired-end reads per sample were generated. Transcript abundance was estimated with Kallisto (29) and differentially expressed (DE) genes were identified using DeSeq2 (30) R package and a FDR corrected p-value <0.05. Gene set enrichment analysis (GSEA) was applied in order to identify gene sets and pathways that were significantly perturbed across conditions. Collections of gene sets were downloaded from MiSigDB (*http://www.broadinstitute.org/gsea/msigdb/*). We used the Benjamini–Hochberg method to adjust gene set p-values and set 0.1 as the significant threshold. Splicing entropy was calculated on TRA transcripts as described in (31). RNA-Seq data are available under accession number GSE166463.

### Sample Preparation and Proteomic Analyses

Murine TEC for proteomic analyses were obtained from three pools of WT or Aβ^0/0^ mice, three mice per pool (5–6 weeks-old). TEC were isolated, enriched and processed through CD45^+^ cell-depletion as described above. CD45^-^ cell-samples were pelleted and stored at −80°C until use. Detailed descriptions of proteomic analysis, data processing, and interaction network reconstructions are described in the *Materials and Methods* section in this article’s **Supplementary Materials**.

### Autoantigen Array

This analysis was performed at the Genomics & Microarray Core Facility at UT Southwestern Medical Center (USA) on human and murine serum or plasma samples. The Autoantigen array contained 95 autoantigens and eight internal control antigens. Profiling of both IgG and IgM autoantibodies was performed. The autoantibodies binding to the antigens on the array were detected with Cy3 labeled anti-IgG and Cy5 labeled anti-IgM and the arrays were scanned with GenePix® 4400A Microarray Scanner. The images were analyzed using GenePix 7.0 software to generate GPR files. The averaged net fluorescent intensity (NFI) of each autoantigen was normalized to internal controls (IgG or IgM).

### Induction of Colitis by Adoptive T-Cell Transfer

CD4^+^ T cells were isolated from pooled spleens and MLNs of 9–10 wk-old WT or Aβ^0/0^ mice as described above. Enriched CD4^+^ T-cell samples were subsequently sorted into naive CD4^+^CD25^−^CD45RB^hi^ and regulatory CD4^+^CD25^hi^CD45RB^−^ cell populations (>98% purity) using a FACS Aria Fusion (Becton Dickinson) cell sorter (FRACTAL facility of San Raffaele Hospital, Milan, Italy). For colitis induction, 4 × 10^5^ WT CD4^+^CD25^−^CD45RB^hi^ naive T cells were transferred by intraperitoneal injection (i.p.) into 8–9 week-old *Rag1^−/−^* mice alone or with 1.5 × 10^5^ CD4^+^CD25^hi^ Treg cells from WT or Aβ^0/0^ mice. Recipient mice were weighted twice a week and sacrificed at week 4 or 8 after the transfer, when colitis was diagnosed by severe weight loss and/or wasting diarrhea. Gut tissues were isolated and analyzed as described and the colon length was measured at time of sacrifice.

### Flow Cytometric Analysis on Human PBMC

PBMC were isolated from PB of HD and patients by density gradient centrifugation using LymphoprepTM (density: 1.077 g/ml; STEMCELL Technologies, Vancouver, Canada). PBMC were maintained in RPMI medium (CORNING) with 2% FBS, 2% L-glutamine, 1% Penicillin/Streptomycin at 4 °C until use or live frozen in FBS + dimethyl sulfoxide (DMSO) 10%. Multi-color immunophenotype of T-lymphocyte subsets on human PBMC was performed by flow cytometry. Details about the monoclonal antibodies used for each staining are reported in the *Materials and Methods* section in this article’s **Supplementary Materials**.

### ELISA Assay

Levels of B-cell activating factor (BAFF) were measured in duplicate in serum or plasma samples of MHCII-D patients and age-matched HD using a Quantikine Human BAFF/BLyS/TNFSF13B Immunoassay kit (R&D Systems, Minneapolis, USA). The assay was performed according to manufacturer’s instructions and the optical density (OD) was determined using a microplate reader.

### Statistical Analyses

All results are expressed as median and interquartile range if not stated otherwise. In the present study a descriptive statistical analysis has been performed. No formal inference was performed due to the small sample size. Statistical significance was assessed using a two-tailed Mann–Whitney test to compare continuous outcomes between groups. T test was used for splicing entropy result analysis. Levels of significance were defined as p ≤0.05 (*), p <0.01 (**), p <0.001 (***), and p <0.0001 (****). Statistical testing was performed using Prism GraphPad (Version 5.0f, La Jolla, CA). Graphs were created using the same software.

## Results

### Thymic Structure Perturbation in Absence of MHC Class II Expression in a MHCII-D Patient and in Aβ^0/0^ Mice

Data about human thymic histology in patients with MHCII deficiency are limited because of the difficulties in accessing these tissues for technical and ethical reasons. In this study, we had the unique opportunity to perform histological analysis of a thymic biopsy performed in a 23-month-old MHCII-D female patient before HSCT (see **Table 1** for immunological data). The patient carried a homozygous mutation in the *CIITA* gene [c.3317 + 2dup], resulting in an intronic splice variant.

**Figure 1:**
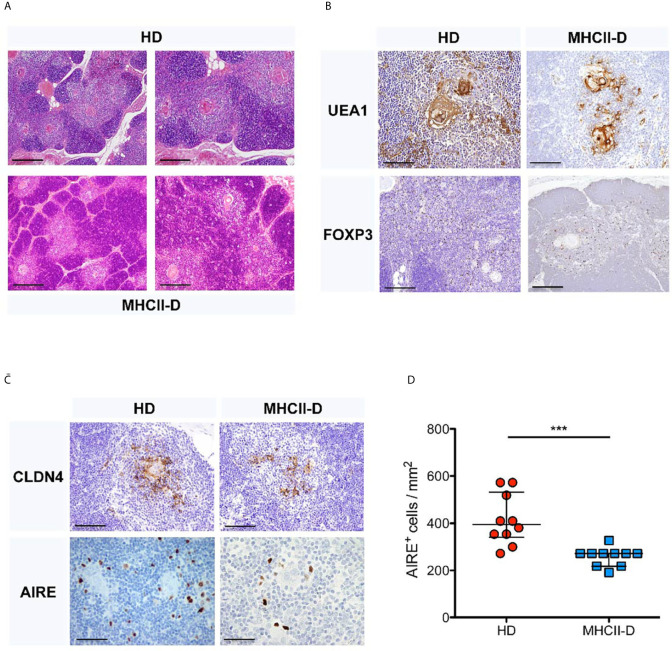
Thymic structure perturbation in a MHCII-D patient’s thymus. **(A)** Histological analysis of a MHCII-D patient’s thymus. Immunohistochemistry analysis of thymic tissue isolated from a human healthy donor (HD) and a MHCII-D patient. Hematoxylin–eosin (H&E) staining shows a reduced representation of thymic medulla. Original magnification 4×, corresponding to 500 μm. **(B)** UEA1 and FOXP3 staining in MHCII-D thymus. Immunohistochemistry analysis of Ulex Europaeus Agglutinin I (UEA1) and FOXP3 expression in a thymic tissue isolated from a human HD and a MHCII-D patient. Original magnifications: 20× for UEA1 and 10x for FOXP3 staining images, corresponding respectively to 100 and 200 μm. **(C)** AIRE and CLDN4 expression in MHCII-D thymus. Immunohistochemistry analysis of CLDN4 (Claudin 4) and AIRE expression in a thymic tissue isolated from a human HD and a MHCII-D patient. Original magnifications: 20× for CLDN4 and 40× for AIRE staining images, corresponding respectively to 100 and 50 μm. **(D)** AIRE expression is reduced in MHCII-D thymus. Comparison of the concentration of AIRE^+^ cells per square millimeter (mm^2^) in a thymic tissue isolated from a human HD and a MHCII-D patient. Mann–Whitney test, p-value < 0.001. Bars represent median with interquartile range. ***p value < 0.001.

The thymic architecture was perturbed, with reduced representation of thymic medulla (**Figure 1A**), as compared to a control thymus analyzed in parallel. This finding was also supported by staining for Ulex Europaeus Agglutinin I (UEA1) and FOXP3 (**Figure 1B**). Moreover, the MHCII-D thymus presented a reduced frequency of AIRE^+^ and CLAUDIN 4 (Cldn4)-expressing TEC, suggesting possible defects of central tolerance (**Figures 1C, D**).

**Figure 2:**
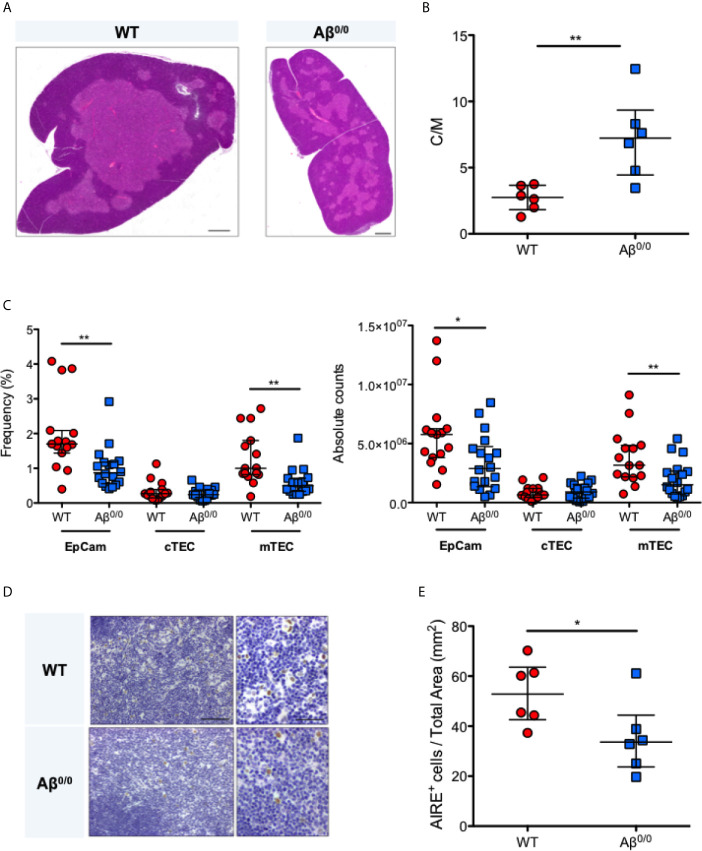
Thymic structure perturbation in Aβ^0/0^ mice thymus. **(A)** Reduced thymic medulla representation in Aβ^0/0^ mice, confirmed by H&E staining as compared to WT mice. Scale bars correspond to 500 μm. **(B)** Increased cortico-medullary (C/M) ratio in Aβ^0/0^ mice. Mann–Whitney test, **p-value <0.01. Median with interquartile range is showed in the graph for each experimental group. **(C)** Reduced frequency (*left panel*) and absolute count (*right panel*) of TEC and mTEC in Aβ^0/0^ mice after enzymatic digestion of thymic tissue and depletion of CD45^+^ cells. Mann–Whitney test, *p value < 0.05; **p value <0.01. **(D)** Immunohistochemical analysis of AIRE expression in WT and Aβ^0/0^ mice thymi. Original magnification: 20× for left panel and 40× for right panel for each condition, corresponding respectively to 100 and 50 μm. **(E)** Frequency of AIRE-expressing cells (AIRE^+^) is reduced in Aβ^0/0^ thymus. Comparison of the concentration of AIRE^+^ cells per mm^2^ of total thymic tissue isolated from WT or Aβ^0/0^ mice. Mann–Whitney test, *p-value < 0.05. Median with interquartile range is showed in the graph for each experimental group.

To further corroborate these observations, we analyzed thymic tissue isolated from the MHCII knock out mouse model, Aβ^0/0^ (17, 18). Histological analysis showed similar abnormalities of thymic architecture, with reduced representation of TECs, especially in the medulla, resulting in a significantly increased cortico-medullary (C/M) ratio in Aβ^0/0^ mice, as compared to WT mice (p-value 0.0087) (**Figures 2A, B**). However, CK5, a medulla specific marker, and CK8 staining indicated a correct compartmentalization of cortical and medullary areas in the thymus of Aβ^0/0^ mice (**Supplementary Figure 1A**). Overall reduced total Epcam^+^ TEC and mTEC frequency and absolute count in Aβ^0/0^ thymi, as compared to WT, were confirmed by flow cytometric analysis (**Figure 2C** and **Supplementary Figure 1B**). Finally, we detected a decreased frequency of AIRE^+^ TEC and FOXP3^+^ cells in the medullary area of Aβ^0/0^ mice thymus, as compared to WT mice (**Figures 2D, E** and **Supplementary Figure 1A**).

### Impaired Generation and Maturation of CD4^+^ SP Thymocytes in Aβ^0/0^ Mice and MHCII-D Patients 

**Figure 3:**
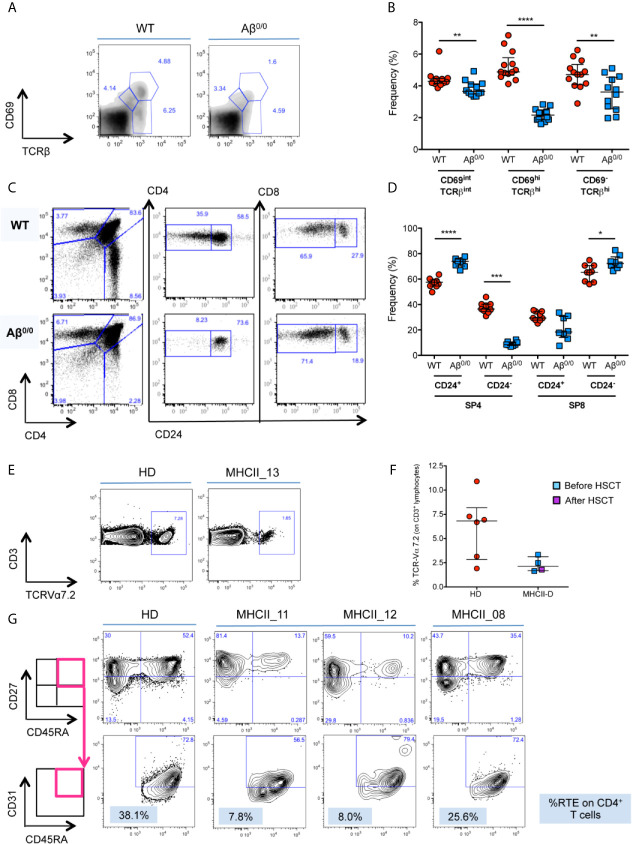
Defective thymopoiesis in MHCII deficiency. **(A)** FACS analysis of thymocytes isolated from WT and Aβ^0/0^ mice, in terms of CD69 and TCRβ expression, shows an impaired positive selection of Aβ^0/0^ thymocytes. Analysis performed on total thymocyte gate. **(B)** Graph shows the summary of all mice analyzed, showing a significant reduction in the frequency of CD69^hi^TCRβ^hi^ post-positive selection thymocytes in Aβ^0/0^ mice, as compared to WT. **p value < 0.01; ***p value < 0.001. **(C)** FACS analysis of thymocytes isolated from WT and Aβ^0/0^ mice shows a normal development of CD4^−^CD8^−^ double negative (DN) subsets. However, Aβ^0/0^ mice CD4^+^ SP cells (SP4) mostly present an immature phenotype (CD24^+^), since only few of them are CD24^−^, as compared to WT SP4 cells. **(D)** Graph shows the summary of all mice analyzed in terms of frequency of CD24^+^ and CD24^−^ SP4 and SP8 cells. *p value < 0.05; ***p value < 0.001; ****p value < 0.0001. **(E, F)** TCR Vα7.2 expression on CD3^+^ T cells is reduced in MHCII-D patients. **(E)** Representative FACS plots on Vα7.2 expression on CD3^+^ T cells on a healthy control (HD) and a MHCII-D patient (MHCII_13). **(F)** Graph shows the summary of all MHCII-D patients analyzed (n = 4) and the healthy controls (HD) tested in parallel. Median and interquartile range are represented for each group. **(G)** Representative FACS plots of CD4^+^ naïve T cells and recent thymic emigrants (RTE) of a healthy control (HD) and three MHCII-D patients, two before HSCT (MHCII_11 and _12) and one after HSCT (MHCII_08). CD27^+^CD45RA^+^ naïve T cells are gated on CD4^+^CD3^+^CD45^+^ T-cell gate, while CD31^+^ RTE are gated on CD27^+^CD45RA^+^ naïve T cell gate.

In accordance with previous reports (17, 18, 24), we observed a reduction in the frequency and in the absolute count of CD69^hi^TCRβ^hi^ thymocytes (**Figures 3A, B** and **Supplementary Figure 2A**), and severe defects of CD4^+^ SP thymocyte generation and maturation (**Figures 3C, D** and **Supplementary Figure 2B**) in Aβ^0/0^ mice, in line with the known fundamental role of MHCII molecules for the progression from the DP stage to the SP CD4^+^ T cell stage (18). Moreover, we confirmed that that majority of these residual SP CD4^+^ thymocytes in Aβ^0/0^ mice corresponds to CD1-restricted NKT cells, as previously described (20, 21) (**Supplementary Figure 2C**). This resulted in peripheral CD4^+^ T cell lymphopenia, with significant reduction in the frequency and absolute count of CD4^+^ naïve T cells and increased frequency of activated CD4^+^ T cells in secondary lymphoid organs (spleen and lymph nodes), as compared to WT CD4^+^ SP cells (SP4) cells (**Supplementary Figures 2D, E**).

Since we could not get access to thymocytes from MHCII-D patients, we investigated TRAV1 (Vα7.2) expression on patients’ PB CD3^+^ lymphocytes as a surrogate marker of thymocyte maturation and survival (32). Interestingly, we found that all four patients analyzed (three before and one after HSCT) showed a trend towards reduced expression of Vα7.2 on their CD3^+^ T cells as compared to normal controls tested in parallel (median: MHCII-D 2.1%; HD 6.8%) (**Figures 3E, F**).

### Reduced Frequency of Naïve CD4^+^ T-Cells and RTE in Untreated MHCII-D Patients

In order to evaluate thymic output in MHCII deficiency, we analyzed the proportion of naïve T cells and recent thymic emigrants (RTE) among PBMC from three patients: two untransplanted [of whom one infant (MHCII_11) and one adult (MHCII_12)], and one transplanted patient (MHCII_08). The percentage of naïve CD45RA^+^CD27^+^CD4^+^ T cells was reduced in the two untreated patients, as compared to the normal donor tested in parallel. Conversely, naïve CD4^+^ T cells in the transplanted patient tended to normal levels (**Figure 3G**). No significant differences emerged in the proportion of naïve CD8^+^ T cells (*data not shown*). We identified RTE as CD31^+^ cells within naïve CD27^+^CD45RA^+^CD4^+^ T cells. In line with data on naïve CD4^+^ cells, the frequency of RTE in the two untransplanted patients was also reduced (**Figure 3G**). This finding was particularly striking for the infant patient. RTE in MHCII_08, who underwent transplant, were comparable to the adult control tested in parallel (**Figure 3G**).

### TEC Transcriptome and Proteome Perturbation in the Absence of MHC Class II

Based on our observations of TEC abnormalities in histologic analysis of thymic samples, we evaluated gene expression profile of Aβ^0/0^ mouse TEC subsets by performing bulk RNA-Seq on sorted WT and Aβ^0/0^ cTEC and mTEC. In order to obtain a sufficient amount of sorted TEC, we pooled 5–10 WT or Aβ^0/0^ mice of 4–6 weeks of age for each biological replicate (n = 3, **Supplementary Table 2**). The gating strategy used to sort cTEC (Epcam^+^CD45^−^Ly51^+^UEA1^−^ cells) and mTEC (Epcam^+^CD45^−^Ly51^−^UEA1^+^ cells) is shown in **Supplementary Figure 3A**. Principal component analysis (PCA) showed that samples were properly grouped according to genetic background and tissue of origin (**Supplementary Figure 3B**).

**Figure 4:**
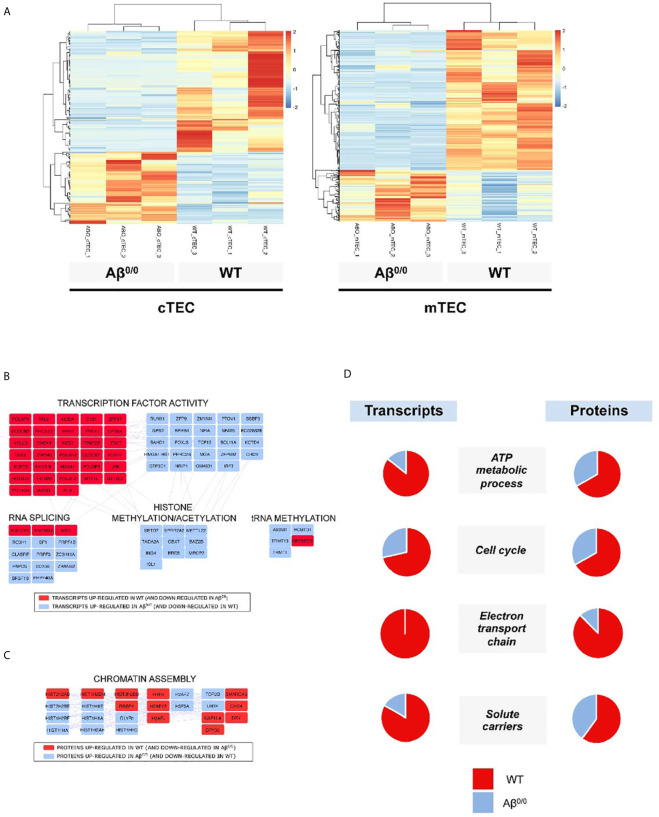
Transcriptome and proteome perturbation in absence of MHCII molecules. **(A)** Heat map of expression values for genes that were differentially expressed between Aβ^0/0^ and WT mice cTEC or mTEC at a p value cut-off (FDR-corrected) of 0.05. Rows (genes) are scaled, i.e., the value (z score) for a gene in a given sample represents its deviation from the mean expression value of the gene across all samples in terms of standard deviations. Up regulation is shown in red, down regulation in blue. Genes and samples are ordered by hierarchical clustering using Pearson’s correlation as the distance measure and complete linkage as the clustering method. **(B)** Network analysis of differentially expressed transcripts in mTEC. The figure shows the main results of network/topology analysis based on the combination of the list of DE transcripts in mTEC with *Mus musculus* Protein–Protein Interaction (PPI) network using STRING bioinformatics tool (full figure is reported in **Supplementary Figure 4A**). In red are represented transcripts up-regulated in WT mTEC, in light blue those up-regulated in Aβ^0/0^ mTEC. **(C)** Cluster-Network analysis of differentially expressed proteins in CD45-depleted TEC fractions. The figure shows the main results of network/topology analysis based on the combination of the list of DE proteins in CD45-depleted TEC fractions with *Mus musculus* Protein–Protein Interaction (PPI) network using STRING bioinformatics tool (full figure is reported in **Supplementary Figure 4B**). In red are represented proteins up-regulated in WT mice, in light blue those up-regulated in Aβ^0/0^ mice. **(D)** Transcript and protein expression per functional cluster in WT and Aβ^0/0^ cell subsets. For each cluster, the proportion of enriched transcripts or proteins in WT (red) or Aβ^0/0^ (blue) are represented as percentage in the respective pie charts. Data about transcripts were obtained from sorted mTEC, data about proteins were obtained from CD45-depleted TEC samples.

Transcriptome analysis revealed an altered gene expression profile in sorted TEC subsets from Aβ^0/0^ mice, as compared to age-matched WT mice. These differences were especially prominent in mTEC (**Figure 4A** and **Supplementary Table 3**), on which we subsequently focused our analyses. In particular, we identified almost one thousand (n = 929) transcripts that were differentially expressed (DE) in mTEC from WT versus Aβ^0/0^ mice. The majority of these transcripts (n = 679, in red in **Supplementary Figure 4A**) were upregulated in mTEC from WT mice (**Supplementary Table 3**). Among DE genes, transcripts that were enriched in Aβ^0/0^ mTEC (n = 250) were mainly relative to RNA splicing, histone modifications and transcription factors (most of which with repressive function) (**Figure 4B**).

Similarly, even if to a lesser extent, cTEC transcriptome resulted also altered (**Figure 4A** and **Supplementary Table 3**) in *Aβ^0/0^*mice. Differentially expressed transcripts upregulated in Aβ^0/0^ cTEC were mainly relative to RNA splicing and translation, carbohydrate metabolism and cytoskeleton. Conversely, in WT cTEC enriched DE transcripts resulted relative to lipid metabolism, peptidases, apoptosis, transmembrane transport, transcriptional regulation, blood vessels/extracellular matrix, cytokine signaling pathway, inflammatory and immune response (**Supplementary Figure 5**).

To further define the cellular features of TEC in Aβ^0/0^ mice, we performed proteomic analysis of bulk CD45-depleted TEC fractions from pools of 4–6-week-old WT and Aβ^0/0^ mice (n = 3, three mice/pool). This analysis could not be performed on sorted TEC subsets due to technical limitations. In these cell subsets, we found 373 DE proteins, 111 of which with high confidence (**Supplementary Tables 4** and **5**). Most of these proteins were upregulated in WT mice (in red in **Supplementary Figure 4B**). Among DE proteins, the only ones that were enriched in Aβ^0/0^ TEC-enriched subsets were relative to chromatin assembly, in particular histones, as indicated by combined cluster-network analysis (**Figure 4C**). We cannot exclude the presence of some fibroblasts and endothelial cells in the analyzed samples.

Next, we performed an integrated network analysis on DE transcripts and proteins in WT and Aβ^0/0^ TEC in order to identify common specific pathways dysregulated in TEC in the absence of MHCII. The only common variation observed in both cTEC and mTEC transcripts and in CD45-depleted TEC fractions was an increased representation of transmembrane transporter activity in WT mice thymi. However, if restricting the analysis to mTEC only, a concordant pattern of variations in both transcripts and proteins within the same cluster between WT and Aβ^0/0^ mice was observed for four clusters (**Figure 4D**). In particular, in WT mice, increased expression was observed for genes and proteins involved in cell metabolism, energy production and cell cycle.

### Impaired Promiscuous Gene Expression in the Absence of MHCII Molecules

**Figure 5:**
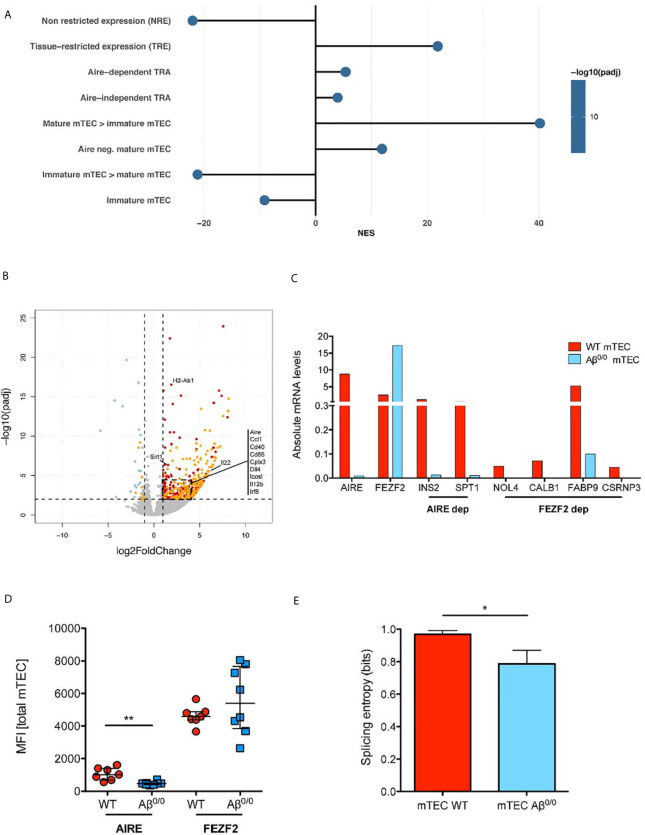
TEC transcriptome perturbation in the absence of MHC class II. **(A)** Enrichment Analysis of Gene Sets involved in promiscuous gene expression and maturation in mTEC. The normalized enrichment score (NES) is reported for each gene set. Positive NES indicate gene sets enriched in WT mTEC, negative NES indicate gene sets enriched in Aβ^0/0^ mTEC. This analysis was performed based on gene lists published in (33). **(B)** Volcano plot representing results of differential gene expression (DE) analysis of genes between WT and Aβ^0/0^ mTEC. In red are represented genes more expressed in WT mTEC, in light blue those more expressed in Aβ^0/0^ mTEC. In orange are evidenced genes with tissue-restricted expression (TRE). **(C)** Absolute mRNA level of Aire, Fefz2, and some TRA (Aire- or Fezf2-dependent), quantified by RT-PCR performed on sorted WT or Aβ^0/0^ mTEC, pooled from 2–3 sorting experiments. Ins2, insulin; Spt1, serine palmitoyltransferase; Nol4, nucleolar protein 4 (testis); Calb1, calbindin (brain, kidney), Fabp9, fatty acid binding protein 9 (testis); Csrnp3, cysteine-serine-rich nuclear protein 3. **(D)** AIRE protein expression is reduced in mTEC lacking MCHII. Graph shows the mean fluorescence intensity (MFI) of AIRE and FEZF2 proteins by flow cytometry in total mTECs after intracellular staining in WT (n = 7) or Aβ^0/0^ (n = 8) mTEC, respectively. **p-value < 0.01. **(E)** Splicing entropy, a measure of the diversity of observed transcripts isoforms in a given sample, resulted decreased in Aβ^0/0^ mTEC. The analysis was restricted to TRA only. The formula used for the calculation of mRNA splicing entropy was derived from (31). Results are expressed as mean and standard deviation. *p value < 0.05.

Next, we interrogated RNA-Seq data for genes known to be involved in the establishment and maintenance of central tolerance. GSEA showed a significant enrichment for both Aire-dependent and Aire-independent transcripts known to have a tissue-restricted pattern of expression, also known as Tissue Restricted Antigen (TRA) genes, in WT mTEC (**Figure 5A**). Reduced expression of TRA genes in Aβ^0/0^ mTEC emerged also at DE analysis and was confirmed by qRT-PCR analysis for a selected number of them (**Figures 5B, C**). Consistent with these observations, *Aire* gene expression was significantly reduced in Aβ^0/0^ mTEC, as compared to WT (Log2 fold change mTEC WT/Aβ^0/0^: 2.12, adjusted p-value: 0.0017) and this result was confirmed by qRT-PCR (**Figures 5B, C**). These results confirm and extend previous observations (24). In addition, we confirmed reduced Aire expression also at the protein level, as shown by intracellular FACS analysis (**Figure 5D**). Analysis of Fezf2 expression did not reveal any relevant differences in Aβ^0/0^ and WT mTEC (**Figures 5C, D**). In addition, DE analysis suggested a disruption of the mechanisms controlling Aire expression in Aβ^0/0^ mTEC, as indicated by a significantly reduced expression of the transcripts encoding for the protein deacetylase Sirtuin-1 (*Sirt1*) and *Irf8* in Aβ^0/0^ mTEC (**Figure 5B**), respectively an essential *Aire* regulator and an *Aire* transcriptional activator. Finally, in order to further investigate mTEC functionality (34), we also calculated TRA splicing entropy (31), a measure of the diversity of transcripts isoforms, which resulted decreased in Aβ^0/0^ mTEC (**Figure 5E**). This disruption of the complex machinery regulating Aire expression could be due to Aβ^0/0^ mTEC impaired maturation. Collectively, these findings suggest that lack of MHCII expression in mTEC is associated with impaired promiscuous gene expression (PGE) and abnormalities of the mechanisms that govern central tolerance.

### Impaired mTEC Maturation in Aβ^0/0^ Mice

RNA-Seq results showed a significantly decreased expression of genes involved in mTEC maturation in Aβ^0/0^ sorted mTEC. Accordingly, GSEA showed a significant enrichment in genes known to be expressed in mature mTEC in WT mice, while genes known to be expressed in immature mTEC where significantly enriched in Aβ^0/0^ mTEC (33) (**Figure 5A**). Moreover, DE gene analysis showed a significantly increased expression of co-stimulatory molecules (mainly CD86 and ICOS ligand, which are involved in TEC-thymocyte cross-talk), in WT mTEC, as compared to their Aβ^0/0^ counterpart (**Figure 5B**). A similar pattern was observed for transcripts encoding for molecules involved in NF-κB signaling, and in particular for CD40, which was more abundantly expressed in WT mTEC (**Figure 5B**). These results were confirmed when analyzing numbers of CD40L^+^ and RANKL^+^ thymocytes in Aβ^0/0^ mice. Indeed, we detected by flow cytometry a severe reduction of both CD40L^+^ and RANKL^+^ thymocyte absolute counts in Aβ^0/0^ mice (**Supplementary Figure 6**), suggesting a low CD40L and RANKL-mediated stimulation of Aβ^0/0^ mTEC. This reduction was particularly evident in mice aged 3 weeks or older (**Supplementary Figure 6**).

### Abnormalities of Peripheral Tolerance in Aβ^0/0^ Mice

**Figure 6:**
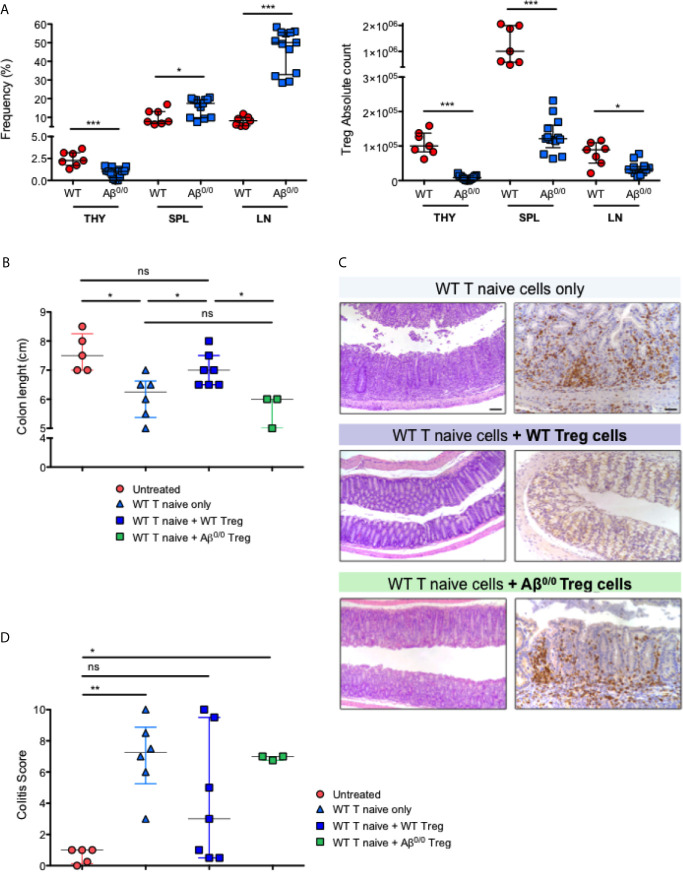
Peripheral tolerance impairment in Aβ^0/0^ mice. **(A)** Frequency and absolute count of Treg cells in WT and Aβ^0/0^ mice. *(Left panel)* Frequency of Treg cells is expressed as the percentage (%) of FoxP3^+^CD25^hi^ cells gated on CD4^+^ T-cell gate. *(Right panel)* Absolute count of Treg cells. THY, thymocytes; SPL, spleen; LN, lymph nodes. *p-value < 0.05; ***p-value < 0.001. **(B)** Colon length in the different treatment groups of induced colitis experiments. The graph shows the colon length of n = 3–7 mice/group from three experiments. Median score is reported for each treatment group. Error bars represent interquartile range. *p-value < 0.05; ns, not significant. **(C)** Histological analysis of the colon of treated mice. Representative colonic sections from Rag1 ko mice who received administration of WT T cells alone or in combination with WT or Aβ^0/0^ Treg cells, stained with H&E (*left panels*) and CD3 immunostaining (*right panels*). Original magnifications: 10× for H&E, 20× for CD3 staining. **(D)** Colitis score in the different treatment groups of induced colitis experiments. The graph shows the inflammation score in the gut of n = 3– mice/group from three experiments. Median score is reported for each treatment group. Error bars represent interquartile range. *p-value < 0.05; **p-value < 0.01; ns, not significant.

Based on the alterations detected in the thymus of MHCII deficient patients and mice, we hypothesized that impairment in central tolerance mechanisms could have an impact also on peripheral tolerance. In line with published results for another MHCII mouse model, the Aα^−/−^ mice (25), Treg cells were nearly absent in the thymus of Aβ^0/0^ mice, but they appeared relatively enriched in spleen and lymph nodes where they were present at higher frequency than in WT mice (**Figure 6A**, left panel). Nonetheless, Treg absolute count was reduced in all lymphoid organs in Aβ^0/0^ mice (**Figure 6A**, right panel).

We then tested in *vivo* the function of Aβ^0/0^ Treg cells. To this purpose, we made use of a model of colitis induced by adoptive transfer of WT T cells, alone or in combination with WT or Aβ^0/0^ Treg cells, into *Rag1* ko mice (**Supplementary Table 6**), and analyzed the capacity of Treg cells to attenuate colitis. Recipient mice were monitored weekly and sacrificed four or eight weeks after the transfer, when signs of colitis (wasting diarrhea and weight loss) became evident in mice that had received WT T naive cells only or in combination with Aβ^0/0^ Treg cells (**Supplementary Figure 7A**). At sacrifice, the gut tissue was analyzed macroscopically and colon length, an indirect sign of gut inflammation (35), was measured. As shown in **Figure 6B**, colon length in mice receiving co-injection of WT Treg cells together with WT T naïve cells, was similar to that of untreated mice. Conversely, colon length was reduced in mice receiving WT naïve T cells alone and in those receiving co-injection of Aβ^0/0^ Treg cells together with WT naïve T cells. Moreover, in mice that received either WT naïve T cells only or in combination with Aβ^0/0^ Treg cells, the study of intestinal pathology revealed different degrees of spontaneous colitis and wasting diarrhea. Substantial thickening of colonic mucosa, indicative of inflammation, was observed. Histologically, colonic inflammation was characterized by crypt elongation and large inflammatory cell infiltrate, mainly consisting of T lymphocytes, with occasional crypt abscesses (**Figure 6C**). A colitis score was calculated on histological sections of gut tissue based on the sum of the evaluation of architectural alterations, degree of inflammation and muciparous gland activity (see *Materials and Methods* for details). When compared to untreated animals, the colitis score was significantly increased in mice receiving WT naïve T cells alone or in combination with Aβ^0/0^ Treg cells, while it was not statistically different in mice receiving both WT naïve T cells and WT Treg cells (**Figure 6D**). Furthermore, among the four treatment groups, only mice that had received co-transplantation of WT Treg cells showed an increased proportion of Treg cells in the spleen and MLN at sacrifice (**Supplementary Figures 7B, C**). Altogether, these results demonstrate that adoptive transfer of Aβ^0/0^ Treg cells failed to block colitis induction in *Rag1* ko mice, suggesting an impaired function of these cells.

### Peripheral Tolerance Impairment in Patients With MHCII Deficiency 

To confirm the results obtained in the mouse model of MHCII deficiency, we set out to evaluate peripheral tolerance in MHCII-D patients. To this purpose, we collected PB samples from a cohort of patients with MHCII deficiency, whose main clinical features are summarized in **Table 2**. *RFXANK* gene mutations were identified in eight of them. Median age at sampling was 4.7 years (range: 0.4–24.1 years). All samples were collected before HSCT, except in one patient for whom only post-transplant sample was available. They suffered from severe and recurrent infections and the majority of them also presented with chronic diarrhea. Autoimmune manifestations were described in three patients. Available data about patients’ immune phenotype and serum immunoglobulin levels are reported in **Table 3**. Most patients were treated with immunoglobulin replacement therapy and anti-infective prophylaxis, as standard supportive care. Seven patients were treated with HSCT, at a median age of 4.5 years (range: 1.1–16.2 years). Characteristics and outcome of HSCT are detailed in **Table 4**.

**Figure 7:**
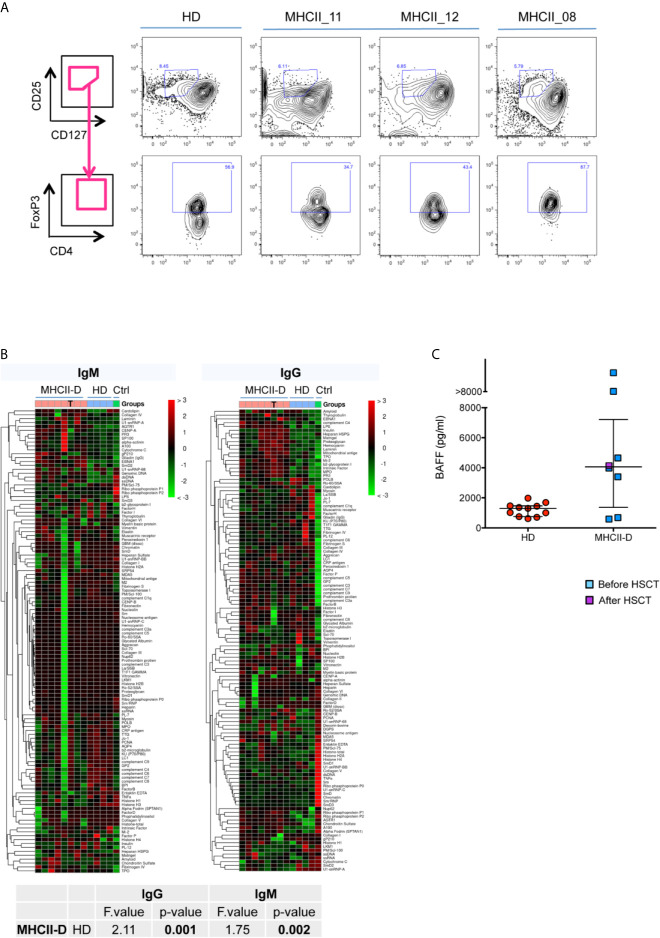
Peripheral tolerance impairment in patients with MHCII deficiency. **(A)** Treg cell frequency in MHCII-D patients. Representative FACS plots of Treg cells in three patients with MHCII-D and a HD. Treg cells are defined as CD4^+^CD25^hi^CD127^lo^FOXP3^+^ cells. In upper panels CD25^hi^CD127^lo^ cells are represented, gated on CD4^+^ T-cell gate. In lower panels, FOXP3^+^ cells are represented, gated on CD25^hi^CD127^lo^ cells. FOXP3 gate was set based on fluorescence-minus-one (FMO) control on normal donor cells. **(B)** Autoantibodies are present in the serum of MHCII-D patients. Patients’ serum was tested for the presence of a large panel of 95 autoantibodies. Heatmap colors correspond to z-score, i.e. the number of standard deviations from the mean signal intensity of the autoantibody across all samples. Color legend over the panel show: pink box, MHCII-D patients; light blue box, age matched normal controls (HD); green, positive control (serum of a patient with systemic lupus erythematous, kindly provided by Stefano Volpi, Genova, Italy). T symbols identify the sample of a MHCII-D patient after HSCT. Statistical analysis included only not transplanted MHCII-D patients. **(C)** BAFF level in MHCII-D patients’ sera. Serum BAFF concentration was determined by ELISA in 11 age-matched healthy controls (HD) and in eight MHCII-D patients (one of whom after HSCT). Each symbol represents an individual, and the median is represented with a horizontal bar. Error bar shows the interquartile range.

We first investigated the frequency of circulating Treg cells in three MHCII-D patients. Treg cells were defined as CD4^+^CD25^hi^CD127^lo^FOXP3^+^ cells. Moreover, in order to differentiate thymic from peripherally induced Treg cells we also stained cells for the expression of HELIOS. The frequency of Treg cells was slightly reduced in one of the untransplanted patients (MHCII_11), as compared to published reference values (36). However, the other two patients showed a frequency of Treg cells comparable to the normal donor, and within normal range also as compared to published references (**Figure 7A** and **Supplementary Table 7**). Interestingly in all patients, most CD4^+^CD25^hi^CD127^lo^FOXP3^+^ cells were also positive for HELIOS, suggesting their likely thymic origin (**Supplementary Table 7**).

We then investigated possible tolerance breakdown as a result of impaired T-B cell cross-talk in the absence of MHCII molecules. In order to evaluate the presence of autoantibodies in the serum of MHCII-D patients, we performed a protein microarray (37) to screen for a large panel of IgG and IgM autoantibodies. This assay revealed a significantly increased titer of many different autoantibodies, especially of IgG isotype, in the serum of MHCII-D patients (**Figure 7B**), as compared to healthy controls. Furthermore, BAFF levels, which are known to play a role in B-cell homeostasis and peripheral tolerance (38, 39), were increased in the sera of most of the patients analyzed, as compared to healthy controls (**Figure 7C**). Both findings of multiple serum autoantibodies and increased BAFF levels, even if not linked to overt autoimmune manifestations in most patients, are suggestive for the presence of a defective peripheral B-cell tolerance checkpoint in MHCII-D.

## Discussion

Our findings in the MHCII deficient mouse model and in human samples from MHCII-D patients suggest that lack of MHCII molecules leads to altered thymic structure and function, resulting in a tolerance impairment broader than previously recognized, involving both central and peripheral mechanisms. In particular, thanks to the unique opportunity to analyze a human thymic biopsy, we observed perturbation of thymic structure in a MHCII-D patient, with reduction of thymic medulla and decreased frequency of AIRE^+^ TEC. These findings are consistent with data obtained in murine models showing thymic structure perturbation with reduced total TEC cellularity and mature mTEC representation. The decreased AIRE^+^ TEC frequency might be caused by medullary area reduction. However, a significantly reduced expression of Aire and Aire-dependent and -independent TRA mRNA by qRT-PCR was previously reported in another MHCII ko mouse model (Aα^−/−^) (24), suggesting also a functional impairment. Whether this apparent functional defect can be explained merely by the reduced size of the thymic medulla, due to impaired thymic cross-talk with developing thymocytes, or by intrinsic TEC defects was not clear. To address this point, we performed an in-depth cellular and molecular characterization of TEC in Aβ^0/0^ mice. Transcriptomic and proteomic studies revealed several differences in the total TEC population and particularly in the mTEC subset, indicative of an overall reduced functionality of these cells in Aβ^0/0^ mice. Interestingly, an integrated network analysis combining RNA-Seq and proteomic data, revealed potential common processes dysregulated in MHCII ko TEC, mainly involving cell metabolism, energy production and cell cycle, that were downregulated in Aβ^0/0^ mice. In line with this, previous studies showed reduced mTEC proliferation capacity in MHCII deficient mice (24). Analysis of PGE revealed profound abnormalities in Aβ^0/0^ mTEC. First, we confirmed known reduced expression of Aire at both mRNA and protein level, while no differences in Fezf2 expression emerged between WT and Aβ^0/0^ mice mTECs. Moreover, further to previous published observations, we extended the analysis on RNASeq data to thousands of genes known to have a tissue-restricted pattern of expression, which resulted significantly enriched in WT mice mTEC, as compared to Aβ^0/0^. Following recent evidence highlighting RNA processing as an additional way to expand the diversity of the self-antigen repertoire displayed by mTEC (31, 34), we tested the capacity of WT and Aβ^0/0^ mTEC to express a great variety of alternatively spliced TRA transcripts. Interestingly, TRA splicing entropy was reduced in Aβ^0/0^ mTEC, suggesting also a qualitative defect in PGE. Various transcription factors and regulators, which associate with AIRE for its expression and function (40), have been shown to modulate PGE. Here we have shown for the first time that two such factors, *Sirt1* and *Irf8*, that have been previously shown to participate at the control of central tolerance induction (41, 42), are expressed at low levels in Aβ^0/0^ mTEC. We have further hypothesized that the abnormalities of PGE in Aβ^0/0^ mice may reflect impaired mTEC maturation. Consistent with this hypothesis, GSEA demonstrated that Aβ^0/0^ mTEC express reduced levels of many genes known to be expressed in mature TEC, including co-stimulatory molecules and key components of the NF-κB pathway, and in particular CD40, on which mTEC developmental program primarily depends on (43).

Thymic cross-talk with developing thymocytes is well-known to a have a fundamental role for the maturation of mTEC, in particular, through RANK-RANKL and CD40-CD40L signaling (24, 43, 44). In line with this, we observed a severe reduction of both CD40L^+^ and RANKL^+^ thymocyte absolute count in Aβ^0/0^ mice. These results support the hypothesis that the underlying cause of reduced mTEC maturation resides in the severe reduction of CD4^+^ thymocytes, resulting in a low RANKL-mediated stimulation of Aβ^0/0^ mTEC, in line with previous data reported by Irla et al. (24). This observation would suggest a possible role for exogenous soluble RANKL administration in overcoming mTEC maturation and number defects in this disease, also based on its beneficial effects on thymic cellularity observed in other disease murine models (45, 46).

We also confirmed impairment of generation and maturation of CD4^+^ SP thymocytes in Aβ^0/0^ mice, which resulted in severe reduction of CD4^+^ T lymphocytes in the periphery, especially in the naïve subset. Defective thymopoiesis in MHCII-D patients has long been considered a reflection of abnormal CD4^+^ SP thymocytes thymic selection and maturation resulting from the absence of MHCII expression on TEC (1, 4, 6, 7, 47), but no specific studies on patients thymocytes have ever been reported due to technical difficulties in accessing these cells in patients. To overcome this limitation, we investigated *TRAV1-2* (Vα7.2) expression on patients’ PB CD3^+^ lymphocytes, which has been recently described to be severely reduced in patients with immunodeficiencies caused by V(D)J recombination and DNA repair defects (32). The evaluation of *TRAV1-2* gene usage allows to indirectly assess the presence of a specific bias in TCRα use, reflecting potential alterations in thymocyte lifespan or alterations in a very specific window of their intrathymic maturation during which thymocytes undergo TCRα rearrangement, the DP stage. Indeed, MHCII molecules are known to have a fundamental role for the progression from this stage to the following SP CD4^+^ stage (18). Interestingly, we found a trend to reduced expression of Vα7.2 on CD3^+^ T cells from the four MHCII-D patients analyzed, including one patient after HSCT. As compared to published mean values obtained in pediatric and adult controls (32), expression of Vα7.2 on their CD3^+^ T cells resulted in the lower end of normal range, suggesting a suboptimal TCRα gene rearrangement in these patients. However, since the expression of Vα7.2 has been shown to be highly heterogeneous (32), both in normal and in general PID population, examining a larger cohort of patients with MHCII-D, both prior and after HSCT, would be needed to confirm this finding. In line with this, previous studies on thymic function in MHCII-D patients reported clonal abnormalities of TCR repertoire and lower TCR gene rearrangement events in patients’ T lymphocytes, as compared to healthy controls (7), suggesting an overall reduced thymic activity in MHCII deficiency and emphasizing the key role of MHCII molecules in the thymic T-cell maturation processes. However, TREC were detected in patients with MHCII deficiency, reflecting normal early T-cell development (4, 7) and published data about naïve CD4^+^ T-cell count in patients with this condition are inconclusive (7). In our study, we observed a reduced frequency of naïve CD45RA^+^CD27^+^CD4^+^ T cells and RTE in the two untreated patients, especially marked in the infant one.

Immune dysregulation is still a poorly characterized feature of MHCII deficiency. Autoimmunity is reported in 6–20% patients (6). The presentation of self-peptides by MHCII molecules is critical for the maintenance of peripheral T-cell tolerance. mTEC support intrathymic generation of Ag-specific FoxP3^+^ Treg cells (48–51), together with negative selection of αβ conventional T cells. Based on the alterations detected at thymic level in the absence of MHCII expression, we hypothesized that impairment in central tolerance mechanisms could impinge also on peripheral tolerance establishment and maintenance, in particular by affecting generation of Treg cells. Previous studies in MHCII ko mice provided evidences in favor of this hypothesis (24). However, it has been previously reported that CD4^+^FoxP3^+^ Treg cells from MHCII ko mice are capable of mediating immune suppression *in vitro* (25). In experimentally induced colitis models, regulatory CD25^+^ DP T cells generated in MHCII ko mice, probably arising from SP CD8^+^ T cells, have been demonstrated to control the colitogenic potential of CD25^-^CD4^+^ T cells (26), but data are missing on the *in vivo* CD4^+^ Treg-specific functionality in MHCII ko models. To address this, we tested Aβ^0/0^ Treg cell capacity to attenuate colitis induced by the injection of WT T naïve cells into *Rag1* ko mice. Our experimental data suggest a functional impairment of Aβ^0/0^ Treg cells *in vivo*, in contrast to previous reports on their *in vitro* functionality. This defect in Aβ^0/0^ mice may be compensated *in vivo* by tolerogenic CD8^+^ “Treg-*like*” cells which have been described to constitutively express CD25, CTLA4 and FoxP3 and have been demonstrated to be able to produce IL-10 and efficiently inhibit CD25^-^ T cell responses to anti-CD3 stimulation (27). These cells might also account, at least in part, for reduced signs of overt spontaneous autoimmunity in Aβ^0/0^ mice.

Limited data regarding Treg cells have been reported to date in patients with MHCII-D. The frequency of circulating Treg cells within the CD4^+^ T-cell population was either normal or slightly reduced in the three patients tested. This is in line with a previously published observation (52) in a pediatric MHCII-D patient, in whom a normal frequency of Treg cells was reported, together with reduced absolute number due to severe reduction in total CD4^+^ T cells in these patients. Of interest, most Treg cells were Helios^+^, suggesting their likely thymic origin.

Impaired Treg function may also contribute to the impairment of peripheral B-cell tolerance checkpoint through altered cognate T-B cell interactions. This is in line with a previous report on a MHCII-D patient who displayed a low number of Treg cells and failed to counterselect autoreactive mature naïve B cells. This suggests that peripheral B-cell tolerance also depends on MHCII-TCR interactions and that Treg cells may play an important role in preventing the accumulation of new emigrant/transitional autoreactive B cells in the mature naive compartment of these patients (52). We found a significantly increased level of BAFF in the serum of most of MHCII-D patients in our cohort. Our results confirm the preliminary observation of increased BAFF levels in one MHCII-D patient reported by Hervé et al. (52), which was correlated with defective peripheral B-cell tolerance checkpoint in this disease. Elevated BAFF levels lower the thresholds for the survival of autoreactive B cell clones (38) and inhibit the counterselection of autoreactive new emigrant/transitional B cells that fail to be removed from B-cell population. It would be important to understand the causes underlying elevation in BAFF level in MHCII-D patients. Elevated BAFF serum levels are often present in B cell lymphopenic conditions (53), autoimmune diseases (54, 55) and viral infections (56). Increased BAFF levels in MHCII-D are unlikely due to peripheral B-cell lymphopenia, since most patients have normal B cell levels, but are more suggestive of ongoing autoreactivity/autoinflammation. BAFF is known to be produced by myeloid cells. In particular, neutrophils can contribute to excess serum BAFF levels, through which they have been demonstrated to promote CD4^+^ T-cell and B-cell responses and enhance CD4^+^ T-cell proliferation and IFNγ secretion in lupus-prone mice (57). Moreover, BAFF has been reported to promote Th1-mediated inflammation through downstream cellular events (58) and to trigger the production of pro-inflammatory cytokines through the activation of the NF-kB pathway (59). These findings indicate a possible link between the chronic infection-driven inflammatory state in these patients and their B-cell tolerance impairment. However, no clear correlation between BAFF levels and autoimmune manifestations emerged from the study of the patients reported here.

In conclusion, our data highlight the key role of MHCII molecules in both central and peripheral immune tolerance mechanisms, and uncover specific defects in TEC maturation and function, both at transcriptomic and proteomic levels. These results also indicate the need of complementing therapeutic approaches based on HSCT with new therapeutic strategies aimed at correcting the underlying molecular defect also at the TEC level in order to achieve a more effective cure for MHCII-D.

## Data Availability Statement

RNA-Seq data are available under accession number GSE166463. Data from proteomic analyses are available in MassIVE repository, at the following link: ftp://massive.ucsd.edu/MSV000086866/. Other raw data supporting the conclusions of this article will be made available by the authors upon request, without undue reservation.

## Ethics Statement

The studies involving human participants were reviewed and approved by the San Raffaele Ethical Committee. Written informed consent to participate in this study was provided by the participants' legal guardian/next of kin. The animal study was reviewed and approved by Institutional Animal Care and Use Committee protocol 710-712.

## Author Contributions

FF performed experiments, analyzed data, and wrote the manuscript. IB performed experiments and analyzed data. RR, GEM, ED, MCC, and GD performed experiments. EF and PLP performed histological analyses on human and murine tissue samples. PU performed RNA-Seq on murine-sorted TEC and data analysis. FB contributed to sample preparation for proteomic studies. DDS performed proteomic and network analyses. DM, CP, TT, VB, ASc, CS, SG, ASo, ARG, SS, BDS, OMD, LDN, and CMR provided patients’ samples and clinical information. LDN, PLP, and PLM contributed intellectual input and data analysis and revised the manuscript. AV and MB designed research experiments, supervised the study, and reviewed the manuscript. All authors contributed to the article and approved the submitted version.

## Funding

This work was supported by the Italian Telethon Foundation (Telethon Core Grant TGT16F03) to MB. FF was supported by a PhD fellowship at Vita-Salute San Raffaele University (Milan, Italy). LDN is supported by the Division of Intramural Research, National Institute of Allergy and Infectious Diseases, National Institutes of Health.

## Conflict of Interest

The authors declare that the research was conducted in the absence of any commercial or financial relationships that could be construed as a potential conflict of interest.

The handling editor declared a shared affiliation with one of the authors (CRM).

## References

[1] Reith W Mach B. The Bare Lymphocyte Syndrome and the Regulation of MHC Expression. Annu Rev Immunol (2001) 19:331–73. 10.1146/annurev.immunol.19.1.331 11244040

[2] Ting JP-Y Trowsdale J. Genetic Control of MHC Class II Expression. Cell (2002) 109:S21–33. 10.1016/S0092-8674(02)00696-7 11983150

[3] Nekrep N Fontes JD Geyer M Peterlin BM. When the Lymphocyte Loses Its Clothes. Immunity (2003) 18:453–7. 10.1016/S1074-7613(03)00086-4 12705848

[4] Hanna S Etzioni A. MHC Class I and II Deficiencies. J Allergy Clin Immunol (2014) 134:269–75. 10.1016/j.jaci.2014.06.001 25001848

[5] Klein C Lisowska-Grospierre B LeDeist F Fischer A Griscelli C. Major Histocompatibility Complex Class II Deficiency: Clinical Manifestations, Immunologic Features, and Outcome. J Pediatr (1993) 123:921–8. 10.1016/S0022-3476(05)80388-9 8229525

[6] Villard J Masternak K Lisowska-Grospierre B Fischer A Reith W. MHC Class II Deficiency: A Disease of Gene Regulation. Med (2001) 80:405–18. 10.1097/00005792-200111000-00006 11704716

[7] Lev A Simon AJ Broides A Levi J Garty BZ Rosenthal E. Thymic Function in MHC Class II-Deficient Patients. J Allergy Clin Immunol (2013) 131:831–9. 10.1016/j.jaci.2012.10.040 23228244

[8] Schuurman HJ van de Wijngaert FP Huber J Schuurman RKB Zegers BJM Roord JJ. The Thymus in “Bare Lymphocyte” Syndrome: Significance of Expression of Major Histocompatibility Complex Antigens on Thymic Epithelial Cells in Intrathymic T-Cell Maturation. Hum Immunol (1985) 13:69–82. 10.1016/0198-8859(85)90014-X 3874194

[9] Ochs H Edvard Smith C Puck JM. Primary Immunodeficiency Diseases – A Molecular and Genetic Approach. 3rd ed. New York: Oxford University Press (2014).

[10] Lum SH Neven B Slatter MA Gennery AR. Hematopoietic Cell Transplantation for MHC Class II Deficiency. Front Pediatr (2019) 7:516. 10.3389/fped.2019.00516 31921728 PMC6917634

[11] Elhasid R Etzioni A. Major Histocompatibility Complex Class II Deficiency: A Clinical Review. Blood Rev (1996) 10:242–8. 10.1016/S0268-960X(96)90008-9 9012922

[12] Posovszky C Sirin M Jacobsen E Lorenz M Schwarz K Schmidt-Choudhury A. Persisting Enteropathy and Disturbed Adaptive Mucosal Immunity Due to MHC Class II Deficiency. Clin Immunol (2019) 203:125–33. 10.1016/j.clim.2019.04.012 31028919

[13] Saleem MA Arkwright PD Davies EG Cant AJ Veys PA. Clinical Course of Patients With Major Histocompatibility Complex Class II Deficiency. Arch Dis Child (2000) 83:356–9. 10.1136/adc.83.4.356 PMC1718526 10999878

[14] Renella R Picard C Neven B Ouachée-Chardin M Casanova JL Le Deist F. Human Leucocyte Antigen-Identical Haematopoietic Stem Cell Transplantation in Major Histocompatiblity Complex Class II Immunodeficiency: Reduced Survival Correlates With an Increased Incidence of Acute Graft-Versus-Host Disease and Pre-Existing Viral Infec. Br J Haematol (2006) 134:510–6. 10.1111/j.1365-2141.2006.06213.x 16848795

[15] Ouederni M Vincent QB Frange P Touzot F Scerra S Bejaoui M. Major Histocompatibility Complex Class II Expression Deficiency Caused by a RFXANK Founder Mutation: A Survey of 35 Patients. Blood (2011) 118:5108–18. 10.1182/blood-2011-05-352716 21908431

[16] Lum SH Anderson C McNaughton P Engelhardt KR MacKenzie B Watson H. Improved Transplant Survival and Long-Term Disease Outcome in Children With MHC Class II Deficiency. Blood (2020) 135:954–73. 10.1182/blood.2019002690 31932845

[17] Cosgrove D Gray D Dierich A Kaufman J Lemeur M Benoist C. Mice Lacking MHC Class II Molecules. Cell (1991) 66:1051–66. 10.1016/0092-8674(91)90448-8 1909605

[18] Grusby MJ Johnson RS Papaioannou VE Glimcher LH. Depletion of CD4+ T Cells in Major Histocompatibility Complex Class II-Deficient Mice. Science (1991) 253:1417–20. 10.1126/science.1910207 1910207

[19] Vanhecke D Verhasselt B De Smedt M De Paepe B Leclercq G Plum J. MHC Class II Molecules Are Required for Initiation of Positive Selection But Not During Terminal Differentiation of Human CD4 Single Positive Thymocytes. J Immunol (1997) 158:3730–7. 9103437

[20] Cardell S Tangri S Chan S Kronenberg M Benoist C Mathis D. CD1-Restricted CD4+ T Cells in Major Histocompatibility Complex Class II-Deficient Mice. J Exp Med (1995) 182:993–1004. 10.1084/jem.182.4.993 7561702 PMC2192275

[21] Waldburger JM Rossi S Hollander GA Rodewald HR Reith W Acha-Orbea H. Promoter IV of the Class II Transactivator Gene Is Essential for Positive Selection of CD4+ T Cells. Blood (2003) 101:3550–9. 10.1182/blood-2002-06-1855 12506036

[22] Nasreen M Ueno T Saito F Takahama Y. *In Vivo* Treatment of Class II MHC-Deficient Mice With Anti-TCR Antibody Restores the Generation of Circulating Cd4 T Cells and Optimal Architecture of Thymic Medulla. J Immunol (2003) 171:3394–400. 10.4049/jimmunol.171.7.3394 14500633

[23] Irla M Guerri L Guenot J Sergé A Lantz O Liston A. Antigen Recognition by Autoreactive CD4+ Thymocytes Drives Homeostasis of The Thymic Medulla. PloS One (2012) 7:1–12. 10.1371/journal.pone.0052591 PMC3531460 23300712

[24] Irla M Hugues S Gill J Nitta T Hikosaka Y Williams IR. Autoantigen-Specific Interactions With CD4+Thymocytes Control Mature Medullary Thymic Epithelial Cell Cellularity. Immunity (2008) 29:451–63. 10.1016/j.immuni.2008.08.007 18799151

[25] Bochtler P Wahl C Schirmbeck R Reimann J. Functional Adaptive CD4 Foxp3 T Cells Develop in MHC Class II-Deficient Mice. J Immunol (2006) 177:8307–14. 10.4049/jimmunol.177.12.8307 17142726

[26] Krajina T Leithäuser F Reimann J. MHC Class II-independent Cd25+ CD4+ Cd8α β+ αβ T Cells Attenuate CD4+ T Cell-Induced Transfer Colitis. Eur J Immunol (2004) 34:705–14. 10.1002/eji.200324463 14991600

[27] Bienvenu B Martin B Auffray C Cordier C Becourt C Lucas B. Peripheral CD8+CD25+ T Lymphocytes From MHC Class II-Deficient Mice Exhibit Regulatory Activity. J Immunol (2005) 175:246–53. 10.4049/jimmunol.175.1.246 15972655

[28] Comans-Bitter WM de Groot R van den Beemd R Neijens HJ Hop WC Groeneveld K. Immunophenotyping of Blood Lymphocytes in Childhood. Reference Values for Lymphocyte Subpopulations. J Pediatr (1997) 130:388–93. 10.1016/S0022-3476(97)70200-2 9063413

[29] Bray NL Pimentel H Melsted P Pachter L. Near-Optimal Probabilistic RNA-seq Quantification. Nat Biotechnol (2016) 34:525–7. 10.1038/nbt.3519 27043002

[30] Love MI Huber W Anders S. Moderated Estimation of Fold Change and Dispersion for RNA-Seq Data With Deseq2. Genome Biol (2014) 15:1–21. 10.1186/s13059-014-0550-8 PMC4302049 25516281

[31] Keane P Ceredig R Seoighe C. Promiscuous mRNA Splicing Under the Control of AIRE in Medullary Thymic Epithelial Cells. Bioinformatics (2015) 31:986–90. 10.1093/bioinformatics/btu785 25429061

[32] Berland A Rosain J Kaltenbach S Allain V Mahlaoui N Melki I. Promidisα: A T-Cell Receptor α Signature Associated With Immunodeficiencies Caused by V(D)J Recombination Defects. J Allergy Clin Immunol (2019) 143:325–34. 10.1016/j.jaci.2018.05.028 29906526

[33] Sansom SN Shikama-Dorn N Zhanybekova S Nusspaumer G Macaulay IC Deadman ME. Population and Single-Cell Genomics Reveal the Aire Dependency, Relief From Polycomb Silencing, and Distribution of Self-Antigen Expression in Thymic Epithelia. Genome Res (2014) 24:1918–31. 10.1101/gr.171645.113 PMC4248310 25224068

[34] Danan-Gotthold M Guyon C Giraud M Levanon EY Abramson J. Extensive RNA Editing and Splicing Increase Immune Self-Representation Diversity in Medullary Thymic Epithelial Cells. Genome Biol (2016) 17:1–13. 10.1186/s13059-016-1079-9 27776542 PMC5078920

[35] Ostanin DV Bao J Koboziev I Gray L Robinson-Jackson SA Kosloski-Davidson M. T Cell Transfer Model of Chronic Colitis: Concepts, Considerations, and Tricks of the Trade. Am J Physiol Gastrointest Liver Physiol (2009) 296:G135–46. 10.1152/ajpgi.90462.2008 PMC2643911 19033538

[36] Arismendi M Kallas E Santos B Carneiro-Sampaio M Kayser C. Thymopoiesis and Regulatory T Cells in Healthy Children and Adolescents. Clinics (2012) 67:425–9. 10.6061/clinics/2012(05)04 PMC3351266 22666784

[37] Rosenberg JM Utz PJ. Protein Microarrays: A New Tool for the Study of Autoantibodies in Immunodeficiency. Front Immunol (2015) 6:138. 10.3389/fimmu.2015.00138 25904912 PMC4387933

[38] Cancro MP. Signalling Crosstalk in B Cells: Managing Worth and Need. Nat Rev Immunol (2009) 9:657–61. 10.1038/nri2621 PMC2766863 19704418

[39] Rowland SL Leahy KF Halverson R Torres RM Pelanda R. Baff Receptor Signaling Aids the Differentiation of Immature B Cells Into Transitional B Cells Following Tonic BCR Signaling. J Immunol (2010) 185:4570–81. 10.4049/jimmunol.1001708 PMC2950883 20861359

[40] Abramson J Goldfarb Y. Aire: From Promiscuous Molecular Partnerships to Promiscuous Gene Expression. Eur J Immunol (2016) 46:22–3. 10.1002/eji.201545792 26450177

[41] Herzig Y Nevo S Bornstein C Brezis MR Ben-Hur S Shkedy A. Transcriptional Programs That Control Expression of the Autoimmune Regulator Gene Aire. Nat Immunol (2017) 18:161–72. 10.1038/ni.3638 27941786

[42] Chuprin A Avin A Goldfarb Y Herzig Y Levi B Jacob A. The Deacetylase Sirt1 Is An Essential Regulator of Aire-Mediated Induction of Central Immunological Tolerance. Nat Immunol (2015) 16:737–45. 10.1038/ni.3194 26006015

[43] Akiyama T Shimo Y Yanai H Qin J Ohshima D Maruyama Y. The Tumor Necrosis Factor Family Receptors RANK and CD40 Cooperatively Establish the Thymic Medullary Microenvironment and Self-Tolerance. Immunity (2008) 29:423–37. 10.1016/j.immuni.2008.06.015 18799149

[44] Lopes N Sergé A Ferrier P Irla M. Thymic Crosstalk Coordinates Medulla Organization and T-Cell Tolerance Induction. Front Immunol (2015) 6:365. 10.3389/fimmu.2015.00365 26257733 PMC4507079

[45] Lo Iacono N Blair HC Poliani PL Marrella V Ficara F Cassani B. Osteopetrosis Rescue Upon RANKL Administration to Rankl-/- Mice: A New Therapy for Human RANKL-Dependent Aro. J Bone Miner Res (2012) 27:2501–10. 10.1002/jbmr.1712 22836362

[46] Lopes N Vachon H Marie J Irla M. Administration of RANKL Boosts Thymic Regeneration Upon Bone Marrow Transplantation. EMBO Mol Med (2017) 9:835–51. 10.15252/emmm.201607176 PMC5452038 28455312

[47] Matheux F Villard J. Cellular and Gene Therapy for Major Histocompatibility Complex Class II Deficiency. Physiology (2004) 19:154–8. 10.1152/nips.01462.2003 15143213

[48] Legoux FP Lim JB Cauley AW Dikiy S Ertelt J Mariani TJ. CD4+ T Cell Tolerance to Tissue-Restricted Self Antigens Is Mediated by Antigen-Specific Regulatory T Cells Rather Than Deletion. Immunity (2015) 43:896–908. 10.1016/j.immuni.2015.10.011 26572061 PMC4654997

[49] Aschenbrenner K D’Cruz LM Vollmann EH Hinterberger M Emmerich J Swee LK. Selection of Foxp3+Regulatory T Cells Specific for Self Antigen Expressed and Presented by Aire+Medullary Thymic Epithelial Cells. Nat Immunol (2007) 8:351–8. 10.1038/ni1444 17322887

[50] Wirnsberger G Hinterberger M Klein L. Regulatory T-Cell Differentiation Versus Clonal Deletion of Autoreactive Thymocytes. Immunol Cell Biol (2011) 89:45–53. 10.1038/icb.2010.123 21042335

[51] Cowan JE Parnell SM Nakamura K Caamano JH Lane PJL Jenkinson EJ. The Thymic Medulla is Required for Foxp3 + Regulatory But Not Conventional CD4 + Thymocyte Development. J Exp Med (2013) 210:675–81. 10.1084/jem.20122070 PMC3620359 23530124

[52] Hervé M Isnardi I Ng Y Bussel JB Ochs HD Cunningham-Rundles C. CD40 Ligand and MHC Class II Expression Are Essential for Human Peripheral B Cell Tolerance. J Exp Med (2007) 204:1583–93. 10.1084/jem.20062287 PMC2118633 17562816

[53] Kreuzaler M Rauch M Salzer U Birmelin J Rizzi M Grimbacher B. Soluble BAFF Levels Inversely Correlate With Peripheral B Cell Numbers and the Expression of BAFF Receptors. J Immunol (2012) 188:497–503. 10.4049/jimmunol.1290009 22124120

[54] Groom J Kalled SL Cutler AH Olson C Woodcock SA Schneider P. Association of BAFF/BLyS Overexpression and Altered B Cell Differentiation With Sjögren’s Syndrome. J Clin Invest (2002) 109:59–68. 10.1172/JCI0214121 11781351 PMC150825

[55] Becker-Merok A Nikolaisen C Nossent H. B-Lymphocyte Activating Factor in Systemic Lupus Erythematosus and Rheumatoid Arthritis in Relation to Autoantibody Levels, Disease Measures and Time. Lupus (2006) 15:570–6. 10.1177/0961203306071871 17080911

[56] Fontaine J Chagnon-Choquet J Valcke H Poudrier J Roger M Groups MPHI. And L-TN-PS. High Expression Levels of B Lymphocyte Stimulator (BlyS) by Dendritic Cells Correlate With HIV-Related B-Cell Disease Progression in Humans. Blood (2011) 117:145–55. 10.1182/blood-2010-08-301887 20870901

[57] Coquery CM Wade NS Loo WM Kinchen JM Cox KM Jiang C. Neutrophils Contribute to Excess Serum BAFF Levels and Promote CD4+ T Cell and B Cell Responses in Lupus-Prone Mice. PloS One (2014) 9:e102284. 10.1371/journal.pone.0102284 25010693 PMC4092127

[58] Sutherland A Ng L Fletcher C Shum B Newton R Grey S. BAFF Augments Certain Th1-Associated Inflammatory Responses. J Immunol (2005) 174:5537–44. 10.4049/jimmunol.174.9.5537 15843552

[59] Sasaki Y Derudder E Hobeika E Pelanda R Reth M Rajewsky K. Canonical NF-κb Activity, Dispensable for B Cell Development, Replaces BAFF-Receptor Signals and Promotes B Cell Proliferation Upon Activation. Immunity (2006) 24:729–39. 10.1016/j.immuni.2006.04.005 16782029

