# Supplementary material for: Thymic Epithelial Cell Alterations and Defective Thymopoiesis Lead to Central and Peripheral Tolerance Perturbation in MHCII Deficiency
Source: Front Immunol. 2021 Jun 15;12:669943. doi: 10.3389/fimmu.2021.669943 (PMC8239840; doi:10.3389/fimmu.2021.669943)
Supplement: Supplementary file 1 [file DataSheet_1.docx]

Supplementary Material

Supplemental Material and Methods

***Flow cytometric analysis of murine cells***

Surface staining of murine TEC was performed with anti-CD45 (30-F11), anti-CD326 (EpCam; G8.8), anti-MHCII (M5 114.15.2) (all Biolegend), anti-Ulex-1 (FL 1061, Vector) and anti-Ly51 (6C3, Miltenyi) monoclonal antibodies. Dead cells were excluded from analyses using DAPI (Sigma Aldrich). Intracellular stainings for the detection of FOXP3, AIRE and FEZF2 proteins were performed using anti-FOXP3 (FJK-16s, eBioscience), anti-AIRE (5H12, eBioscience) and anti-FEZF2 (ab69436, Abcam) antibodies on cells after fixation and permeabilization (Cytofix/Cytoperm kit; BD Pharmingen). Alexa Fluor 405 (Invitrogen) secondary antibody was used for detection of anti-FEZF2 primary antibody.

Thymocytes, splenocytes, lymph node derived-cells were incubated with anti-CD45 (30-F11), anti-CD3 (17A2), anti-CD4 (RM 4-5), anti-CD8 (53-6.7), anti-CD25 (PC61), anti-CD24 (M1/69), anti-CD69 (H1.2F3), anti-CD44 (IM7), anti-TCRβ (H57-597), anti-TCR gamma/delta (GL3), anti-CD62L (MEL14) (all BD Pharmingen/Biosciences), anti-CD19 (1D3), anti-RANKL (IK22/5), anti-GITR (DTA-1, eBioscience), anti-MHC II (M5 114.15.2), anti-CD40L (MR1) and anti-CD45RB (C363-16A) (all Biolegend) and streptavidin (Invitrogen by Thermo Fisher Scientific). Discrimination between live and dead cells was performed by LIVE/DEAD Fixable Yellow Dead Cell Stain Kit (Invitrogen by Thermo Fisher Scientific).

In order to analyze iNKT cells in the thymus, thymocytes were incubated in FACS buffer (2% FBS + 0.2% NaN3 in PBS) for 10 minutes in Fc Blocker (Purified Rat anti-mouse CD16/CD32, clone 2.4G2, BD Biosciences), then stained with fluorochrome-conjugated mAb specific for CD24 (FITC, clone M1/69, BioLegend), TCRβ (PE, clone H57-597, BioLegend), CD4 (APC/Cy7, clone RM4-5, BioLegend), NK1.1 (PE/Cy7, clone PK136, BioLegend) and CD1d tetramer (mouse PBS57-CD1d-tetramer APC, provided by National Institute of Health -NIH- tetramer core facility). Dead cells were excluded with DAPI (4’,6-diamidino-2-phenilynole, 100 ng/ml).

***Proteomic analysis***

Murine CD45^-^ TEC samples were lysed through three quick freeze/thaw cycles and the protein extraction was performed by adding RapiGestTM SF at 0.2% (w/w) according to the manufacture’s protocol (Waters Corporation, Milford, MA, USA). Enzymatic digestion was conducted by adding trypsin in a ratio 1:50 (w/w) o/n and then a further aliquot in a ratio 1:100 (w/w) was added for 4 h. Each peptide mixture was desalted by Pep-Clean C-18 spin columns (Pierce Biotechnology Inc., Rockford, IL, USA), concentrated at 60°C and finally reconstituted in 0.1% formic acid. After enzymatic digestion with trypsin, samples were analyzed using nano-chromatography coupled to orbitrap mass spectrometer (LC MS/MS). Globally, 3 biological samples per condition were analyzed in technical replicates (n=2). In particular, the chromatographic separation of peptides was performed using the Eksigent nanoLC-Ultra® 2D System (Eksigent, part of SCIEX Dublin, CA, USA) combined with cHiPLC®- nanoflex system (Eksigent) in trap-elute mode. Peptides were eluted in 100 min with an acetonitrile gradient (mobile phase A: 0.1% formic acid in water; mobile phase A: 0.1% formic acid in acetonitrile), ionized by a nanospray ionization source (EASY-SprayTM 90 Source, Thermo Fisher Scientific, San Josè, CA, USA) and analyzed using QExactive mass spectrometer (Thermo 91 Fisher Scientific, San Josè, CA, USA). Full mass spectra were recorded in positive ion mode in a range of 400-1600 m/z, with a resolution of 70000 FWHM (@ m/z 200) and 1 microscan per second. Each full scan was followed by 7 MS/MS events (resolution of 17,500 FWHM) generated in a data dependent manner on the top seven most abundant isotope patterns with charge ≥ 2 (isolation window of 2 m/z from the survey scan, normalized collision energy of 30 and dynamic exclusion of 30 sec).

*Proteomics Data Processing*

The experimental tandem mass spectra (MS/MS) produced by LC-MS/MS analysis were matched against the *in-silico* tryptic peptide sequences of the *Mus musculus* protein database retrieved from UNIPROT (*www.uniprot.org*). Data processing was performed by Discoverer 2.1 software, based on SEQUEST HT algorithm, as previously reported (1). Specifically, matches between spectra were only retained if they had a minimum Xcorr of 2.0 for +1, 2.5 for +2 and 3.5 for +3 charge state, respectively, protein rank was fixed to 1 while peptide confidence to “*high”.* In addition, the FDR was set to ≤ 5%. The spectral count (SpC) values of the proteins identified were normalized using a Total Signal normalization method (2) and compared by a label-free quantification approach as previously reported (3). Specifically, protein lists were aligned and then processed by means of Linear Discriminant Analysis (LDA) (4) and g-test (5). LDA was applied by using a common covariance matrix for all groups, and the Mahalanobis distance (6) from each point to each group’s multivariate mean. To select proteins discriminating the analysed conditions (WT vs Aβ^0/0^), we considered those with smallest *P*-value (≤ 0.05) and *F* ratio ≥ 3.5. The average Spectral Count (aSpC) values of proteins selected by LDA were pairwise compared by DAve and DCI index of MAProMa software (7); in addition, the fold change was estimated by the natural logarithm of the SpC ratio (SpC1/SpC2). Finally, proteins selected by LDA were evaluated by hierarchical clustering (8) using the Euclidean’s distance metric and Complete method.

Data from proteomic analyses are available in MassIVE repository, at the following link: ftp://massive.ucsd.edu/MSV000086866/.

*Protein-protein interaction network reconstruction*

Proteins selected by LDA were evaluated at functional level and a corresponding *Mus musculus* protein-protein (PPI) interaction network was reconstructed by retrieving interaction from STRING db (9); only interactions experimentally determined or database annotated were considered. To retrieve and visualize interactions, Cytoscape software was used (10), while Cytoscape plugin BINGO 2.44 (11) allowed to evaluate the most represented GO terms (GO Biological Processes, GO Molecular Functions, GO Cellular Component); specifically, *Mus musculus* organism, hypergeometric test, Benjamini & Hochberg FDR correction and a significance level ≤0.05 were applied.

The same approach was applied for the network analysis of differentially expressed transcripts obtained from RNA-Seq analysis of mTEC from WT or Aβ^0/0^ mice.

**Flow cytometric analysis on human PBMC**

To study TCRα rearrangement, we used the following monoclonal antibodies (mAbs) [adapted from (12)]: anti-CD3 APC (BW264/56), anti-CD4 PerCP (VIT4), anti-CD8 VioBlue (BW135/80), anti-CD45 APCH7 (5B1) (all from Milteniy Biotech), and anti-TCR Valfa7.2 PE (3C10, Biolegend).

To identify the naïve/memory T-cell subsets and recent thymic emigrants (RTE), a multi-color staining on frozen PBMC was performed using the following mAbs: anti-CD45 APC (clone 5B1), anti-CD3 VioGreen (REA613), anti-CD4 PE-Vio770 (M-T321), anti-CD8 PE (BW135/80), anti-CD27 VioBlue (M-T271) and anti-CD31 APC-Vio770 (AC128) (all from Miltenyi Biotech), anti-CD45RA PerCP/Cy5.5 (HI100) (BioLegend).

Peripheral Treg cells were analyzed with a seven-color labeling on frozen PBMC using the following mAbs: anti-CD4 PE-Vio770 (clone M-T321), anti-CD45 APC-Vio770 (5B1) (all from Miltenyi Biotech), anti-CD25 APC (M-A251), anti-CD45RA PerCP/Cy5.5 (HI100), anti-CD127 (IL7Rα) PE (A019D5) (BioLegend). The surface staining was followed by the intracellular staining using anti-FoxP3 Alexa Fluor 488 (259D) and anti-Helios Pacific Blue (22F6) (BioLegend) antibodies after fixation and permeabilization (FoxP3 Staining Buffer Set, eBioscience), performed following the protocol recommended by the manufacturer.

**Supplementary References**

1. Sereni L, Castiello MC, Marangoni F, Anselmo A, di Silvestre D, Motta S, Draghici E, Mantero S, Thrasher AJ, Giliani S, et al. Autonomous role of Wiskott-Aldrich syndrome platelet deficiency in inducing autoimmunity and inflammation. *J Allergy Clin Immunol* (2018) **142**:1272–1284. doi:10.1016/j.jaci.2017.12.1000

2. Carvalho PC, Fischer JSG, Chen EI, Yates JR, Barbosa VC. PatternLab for proteomics: A tool for differential shotgun proteomics. *BMC Bioinformatics* (2008) **9**:1–14. doi:10.1186/1471-2105-9-316

3. Vigani G, Di Silvestre D, Agresta AM, Donnini S, Mauri P, Gehl C, Bittner F, Murgia I. Molybdenum and iron mutually impact their homeostasis in cucumber (Cucumis sativus) plants. *New Phytol* (2017) **213**:1222–1241. doi:10.1111/nph.14214

4. Hilario M, Kalousis A. Approaches to dimensionality reduction in proteomic biomarker studies. *Brief Bioinform* (2008) **9**:102–118. doi:10.1093/bib/bbn005

5. Zhang B, VerBerkmoes NC, Langston MA, Uberbacher E, Hettich RL, Samatova NF. Detecting Differential and Correlated Protein Expression in Label-Free Shotgun Proteomics. *J Proteome Res* (2006) **5**:2909–2918. doi:10.1021/pr0600273

6. Jain A, Murty M, Flynn P, Rosenfeld A, Bowyer K, Ahuja N, et al. Data Clustering: A Review. *ACM 216 Comput Surv* (1999) **31**:264–323.

7. Mauri P, Dehò G. “A Proteomic Approach to the Analysis of RNA Degradosome Composition in Escherichia coli.,” in *Methods in enzymology*, 99–117.

8. Zhao Y, Karypis G. Clustering in Life Sciences. *Funct Genomics* (2005) **31**:183–218. doi:10.1385/1-59259-364-X:183

9. von Mering C, Jensen L, Snel B, Hooper S, Krupp M, Foglierini M, Jouffre N, Huynen M, Bork P. STRING: known and predicted protein-protein associations, integrated and transferred across organisms. *Nucleic Acids Res* (2005) **33**:D433-7.

10. Shannon P, Markiel A, Ozier O, Baliga NS, Wang JT, Ramage D, Amin N, Schwikowski B, Ideker T. Cytoscape: A Software Environment for Integrated Models of Biomoleculare Interaction Networks. *Genome Res* (2003) **13**:2498–504. doi:10.1101/gr.1239303.metabolite

11. Maere S, Heymans K, Kuiper M. BiNGO: A Cytoscape plugin to assess overrepresentation of Gene Ontology categories in Biological Networks. *Bioinformatics* (2005) **21**:3448–3449. doi:10.1093/bioinformatics/bti551

12. Berland A, Rosain J, Kaltenbach S, Allain V, Mahlaoui N, Melki I, Fievet A, Dubois d’Enghien C, Ouachée-Chardin M, Perrin L, et al. PROMIDISα: A T-cell receptor α signature associated with immunodeficiencies caused by V(D)J recombination defects. *J Allergy Clin Immunol* (2019) **143**:325–334. doi:10.1016/j.jaci.2018.05.028

**Supplementary Tables**

**Supplementary Table 1. List of the Oligos used to perform RT-PCR on sorted TEC.**

| **RT-PCR on sorted TEC** | | |
| --- | --- | --- |
| Actb | for | 5′-CTAAGGCCAACCGTGAAAAG-3′ |
| Actb | rev | 5′-ACCAGAGGCATACAGGGACA-3′ |
| Aire | for | 5′-CCAGTGAGCCCCAGGTTAAC-3′ |
| Aire | rev | 5′-GACAGCCGTCACAACAGATGA-3′ |
| Fezf2 | for | 5′-GTGGCTCCCACCTTTGTACATTCA-3′ |
| Fezf2 | rev | 5′-TCACGGTGACAGGCTGGGATTAAA -3′ |
| Ins2 | for | 5′-GACCCACAAGTGGCACAA -3′ |
| Ins2 | rev | 5′-ATCTACAATGCCACGCTTCTG -3′ |
| Spt1 | for | 5′-GTGTTGCTTGGTGTTTCCAC -3′ |
| Spt1 | rev | 5′-GCAGAATCAGCAGTTCCAGA -3′ |
| Nol4 | for | 5′-AGGCTCCCAGGAGGTATTGT -3′ |
| Nol4 | rev | 5′-AGCTGCCTCTTCATGCTCAG -3′ |
| Calb1 | for | 5′-AATGAGCTGGATGCTTTGCT -3′ |
| Calb1 | rev | 5′-TCCGACAAGGCCATTATGTT -3′ |
| Fabp9 | for | 5′-AAATGGCTTGGCAAACAGAC -3′ |
| Fabp9 | rev | 5′-CATTGTTCATGGTGCACTCC -3′ |
| Csrnp3 | for | 5′-CTGGCAGGCATTAAGTGTCA -3′ |
| Csrnp3 | rev | 5′-AGTCCGCAGACGGATAGGAT -3′ |

**Supplementary Table 2. Cell yield after sorting.**

The table details the cell yield after each sorting, in terms of total sorted cells, sorted cTEC and mTEC in WT and Aβ^0/0^ mice.

| ***Cell yield after sorting*** | **WT** | **Aβ^0/0^** |  |
| --- | --- | --- | --- |
| Total sorted cells (x10^6^) | 0.94 | 1.49 | **Replicate 1** |
| cTEC | 2600 | 1810 |  |
| mTEC | 19200 | 4750 |  |
| Total sorted cells (x10^6^) | 1.30 | 0.88 | **Replicate 2** |
| cTEC | 6300 | 4300 |  |
| mTEC | 35500 | 6800 |  |
| Total sorted cells (x10^6^) | 1.30 | 0.63 | **Replicate 3** |
| cTEC | 10300 | 2300 |  |
| mTEC | 35000 | 4200 |  |

**Supplementary Table 3. Differentially expressed genes between WT and Aβ^0/0^ TEC subsets (mTEC and cTEC) at RNA-Seq analysis.**

The list of differentially expressed genes is reported in a separate Excel file. Only significantly differentially expressed genes have been reported (FDR corrected p-value < 0.05).

**Supplementary Table 4. Proteins identified in bulk CD45-depleted TEC fractions from WT and Aβ^0/0^ mice.**

The list of identified proteins is reported in a separate Excel file. For each protein, UNIPROT ID, Gene Name, Protein Name, pI, MW and Spectra count (SpC) values are shown.

**Supplementary Table 5. Differentially expressed proteins in bulk CD45-depleted TEC fractions from WT and Aβ^0/0^ mice.**

The list of differentially expressed proteins is reported in a separate Excel file. In bold, high-confidence differentially expressed proteins [selected by LDA (p<0.05) and/or g-test (p<0.05)] are shown. Fold change [LN(SpC_WT/SpC_Aβ^0/0^)] is reported. Positive (red) fold change values indicate protein up-regulated in WT CD45^-^ TEC fractions, while negative (blue) fold change values indicate proteins up-regulated in Aβ^0/0^ CD45^-^ TEC fractions. For proteins identified only in one condition, fold change was set to +/- 5 by default.

**Supplementary Table 6. Induced colitis experiment treatment groups**.

In the table, the total numbers of mice treated in 3 separate experiments are reported. No., number.

| **No. of mice** | **Treatment group** |
| --- | --- |
| 5 | Untreated |
| 6 | WT T naive only |
| 7 | WT T naive **+ WT Treg** |
| 3 | WT T naive **+ Aβ^0/0^ Treg** |

**Supplementary Table 7. Frequency of Treg cells in MHCII-D patients’ peripheral blood.**

Adult normal donor tested in parallel:

- % Treg (on CD4^+^ cells): 4.8%.
- % Helios^+^ Treg cells (on total Treg): 63.6%.

| Patient | Age  (yrs) | Pre/Post HSCT | % Treg  (on CD4^+^cells) | % Helios^+^ Treg |
| --- | --- | --- | --- | --- |
| MHCII_11 | 0.4 | Pre | 2.1 | 90.6 |
| MHCII_12 | 24.1 | Pre | 3.0 | 96.3 |
| MHCII_08 | 6.5 | Post | 5.1 | 89.9 |

**Supplementary Figure legends**

**Supplementary Figure 1**

**A.** Immunohistochemical analysis of WT and Aβ^0/0^ mice thymus. Cytokeratin 5 (CK5), cytokeratin 8 (CK8), UEA1 and FOXP3 staining images are reported. Original magnification: 10x, corresponding to 200 μm.

**B.** Representative FACS plots showing the gating strategy used to discriminate cTEC (Ly51^+^UEA1^-^) and mTEC (Ly51^-^UEA1^+^) among total Epcam^+^CD45^-^ TEC.

**Supplementary Figure 2**

**A.** Absolute counts of developing thymocytes subsets: CD69^int^TCRβ^int^, CD69^hi^TCRβ^hi^ and CD69^-^TCRβ^hi^. ** p value <0.01.

**B.** Absolute counts of maturing SP4 and SP8 thymocytes, according to the presence or absence of CD24 surface marker. ** p value <0.01; **** p value <0.0001.

**C.** CD4^+^ iNKT cell representation in the thymus of WT and *Aβ^0/0^* mice.

**D-E.** CD4^+^ lymphocytes show an activated phenotype in peripheral lymphoid tissues of Aβ^0/0^ mice. Graphs show the summary of all mice analyzed in terms of frequency (*left panels*) and absolute counts (*right panels*) of naïve (CD62L^+^CD44^-^) and activated (CD62L^-^CD44^+^) cells within CD4^+^ and CD8^+^ cell subsets, as resulted from FACS analysis of lymphocytes isolated from spleen **(D)** and lymph nodes **(E)** of WT and Aβ^0/0^ mice. *p value <0.05; ** p value <0.01; *** p value <0.001.

**Supplementary Figure 3**

**A.** TEC subset sorting strategy. cTEC were sorted as Epcam^+^CD45^-^Ly51^+^UEA1^-^ cells, mTEC as Epcam^+^CD45^-^Ly51^-^UEA1^+^ cells.

**B.** Principal component analysis (PCA) of mRNA expression data on sorted TEC. The first 2 principal components (PC) shown explain the largest part of the variation in mRNA expression (PC1, 74%; PC2, 8%). PCA confirmed that replicates behaved similarly.

**Supplementary Figure 4**

**(A)** Network analysis of differentially expressed transcripts in mTEC. Full figure showing the results of network/topology analysis based on the combination of the list of DE transcripts in mTEC with Mus musculus Protein-Protein Interaction (PPI) network using STRING bioinformatics tool. In red are represented transcripts up-regulated in WT mTEC, in jade green those up-regulated in Aβ^0/0^ mTEC.

**(B)** Cluster-Network analysis of differentially expressed proteins in CD45-depleted TEC fractions. Full figure showing the results of network/topology analysis based on the combination of the list of DE proteins in CD45-depleted TEC fractions with Mus musculus Protein-Protein Interaction (PPI) network using STRING bioinformatics tool. In red are represented proteins up-regulated in WT mice, in jade green those up-regulated in Aβ^0/0^ mice.

**Supplementary Figure 5**

Network analysis of differentially expressed transcripts in cTEC. The figure shows the results of network/topology analysis based on the combination of the list of DE transcripts in cTEC with Mus musculus Protein-Protein Interaction (PPI) network using STRING bioinformatics tool. In red are represented transcripts up-regulated in WT cTEC, in jade green those up-regulated in Aβ^0/0^ mTEC.

**Supplementary Figure 6**

CD40L and RANKL expression on WT and Aβ^0/0^ thymocytes. Graph shows the absolute count of CD40L^+^ **(A)** or RANKL^+^ **(B)** CD4^+^TCRβ^hi^ thymocytes in WT and Aβ^0/0^ mice of different ages (3 days, n=3; 3 weeks, n=4; 6 weeks, n=4). Median and interquartile range are represented for each group. *, p-value <0.05.

**Supplementary Figure 7**

**A.** Mice weight course after treatment in induced colitis experiments. This graph is representative of 2 separate experiments. Mean and SD are reported for each time point. Number of mice per group: untreated, n=3; WT T naive only, n=5; WT T naive + WT Treg, n=4; WT T naive + Aβ^0/0^ Treg, n=2.

**B.** Treg cells in mesenteric lymph nodes (MLN) at sacrifice. Representative FACS plot of Treg cells in MLN in the 4 treatment groups. Treg cells are represented by the percentage of CD25^+^GITR^+^cells on CD3^+^CD4^+^ cell gate.

**C.** Treg frequency in secondary lymphoid organs. The graph shows the frequency of Treg (CD25^+^GITR^+^) cells on CD3^+^CD4^+^ T-cell gate of n=3-7 mice/group from 3 experiments. Median is reported for each treatment group. Error bars represent interquartile range. *, p-value <0.05; **, p-value <0.01; ns, not significant.
