# Supplementary figures and images for: Thymic Epithelial Cell Alterations and Defective Thymopoiesis Lead to Central and Peripheral Tolerance Perturbation in MHCII Deficiency
Source: Front Immunol. 2021 Jun 15;12:669943. doi: 10.3389/fimmu.2021.669943 (PMC8239840; doi:10.3389/fimmu.2021.669943)

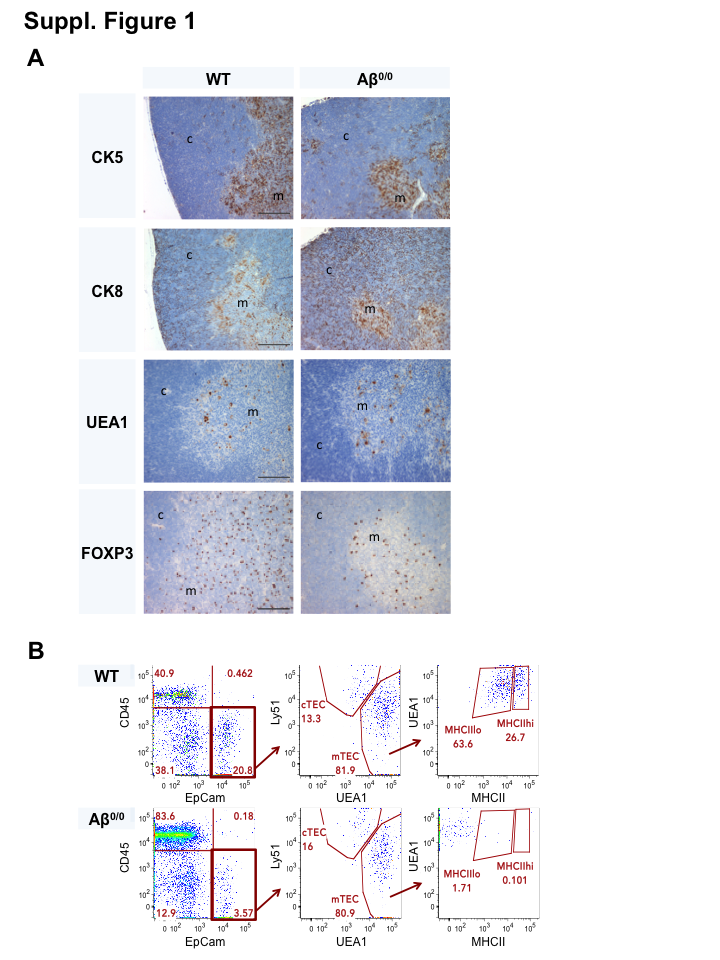

Supplement: Supplementary file 2 [file Image_1.tif]

Suppl. Figure 2

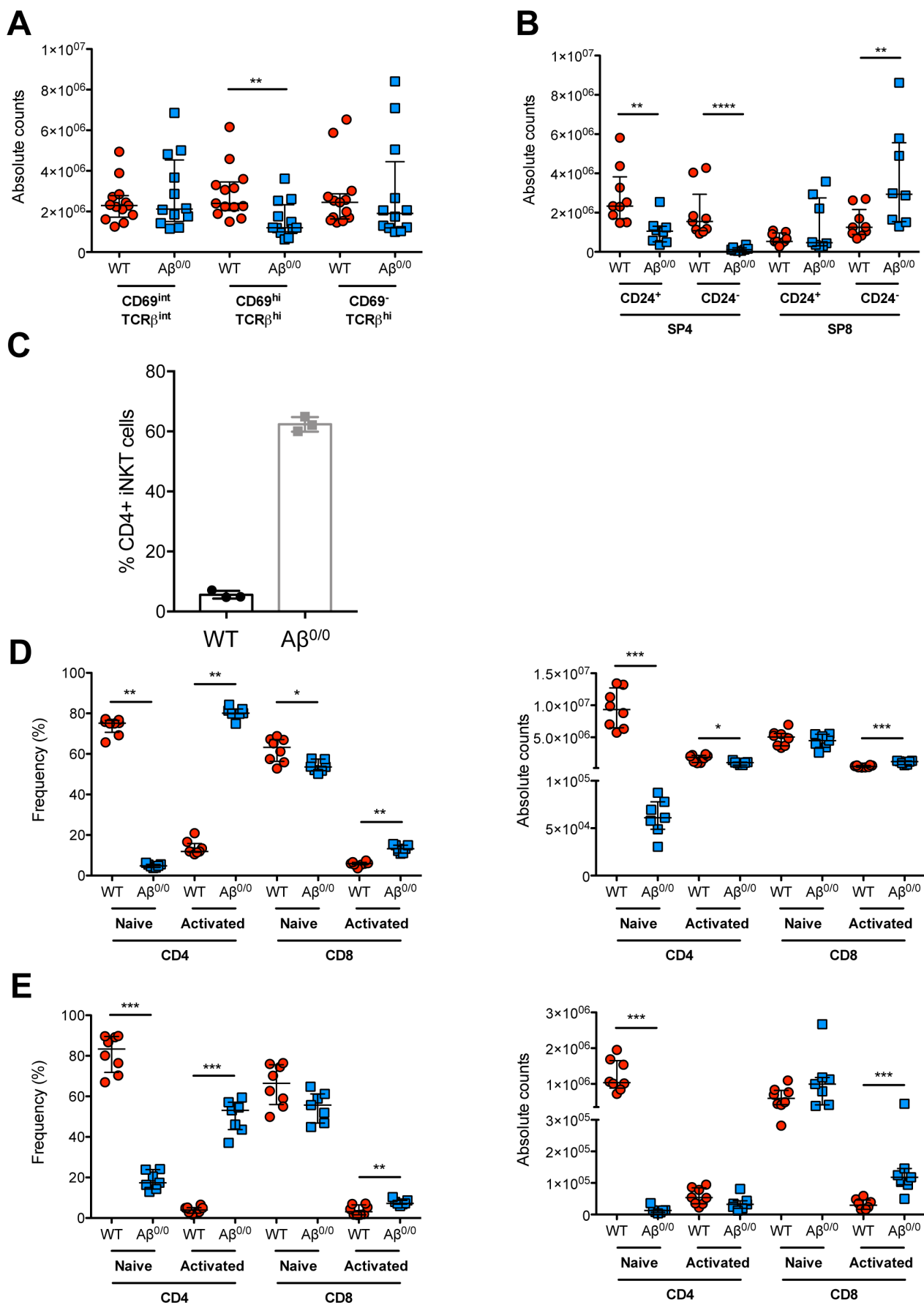

Supplement: Supplementary file 3 [file Image_2.pdf]

Suppl. Figure 3

A

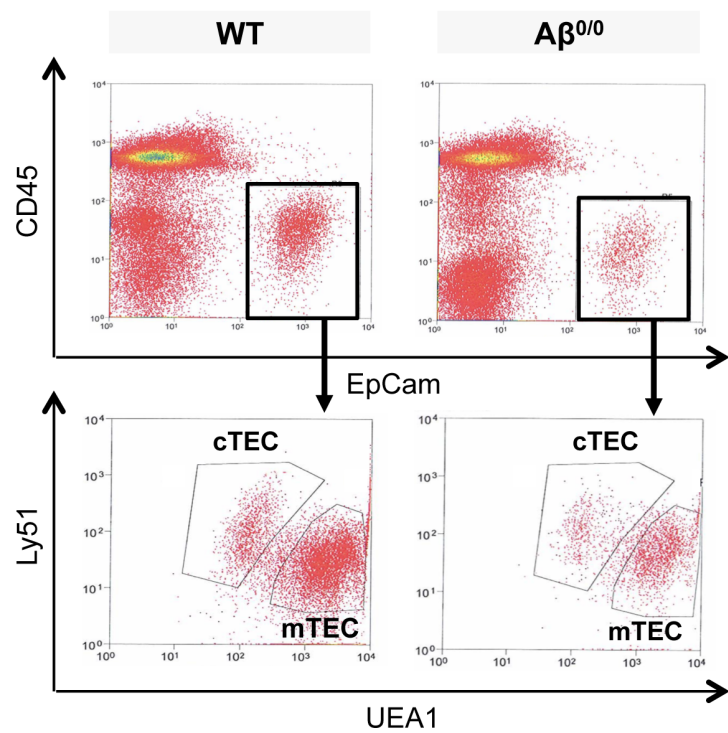

B

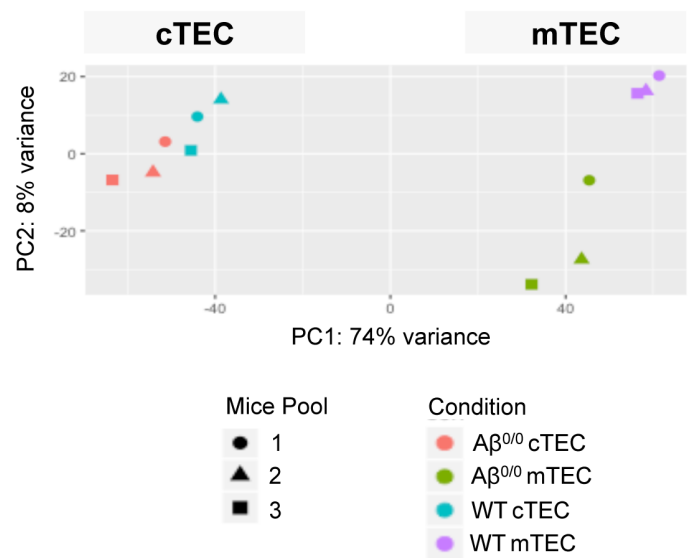

Supplement: Supplementary file 4 [file Image_3.pdf]

# Suppl. Figure 4

A

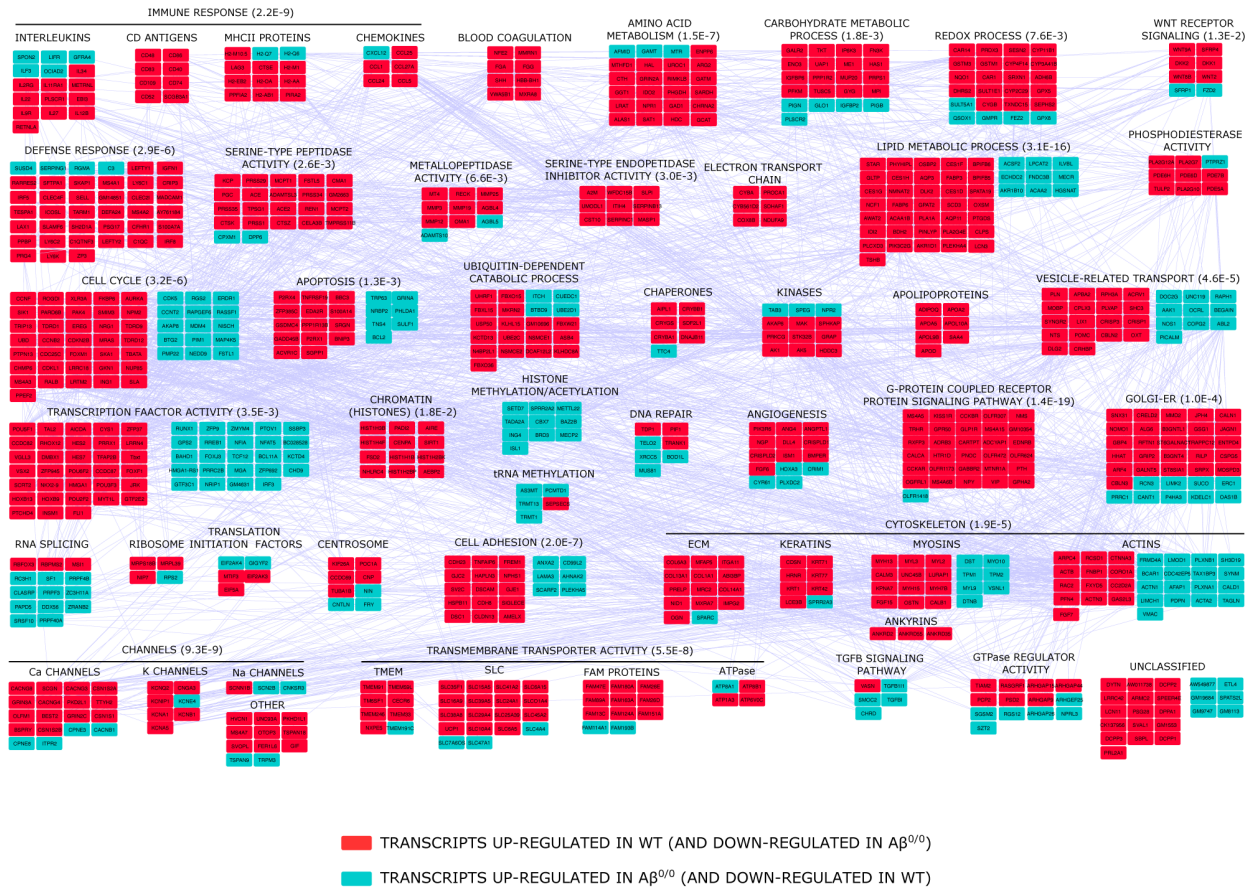

B

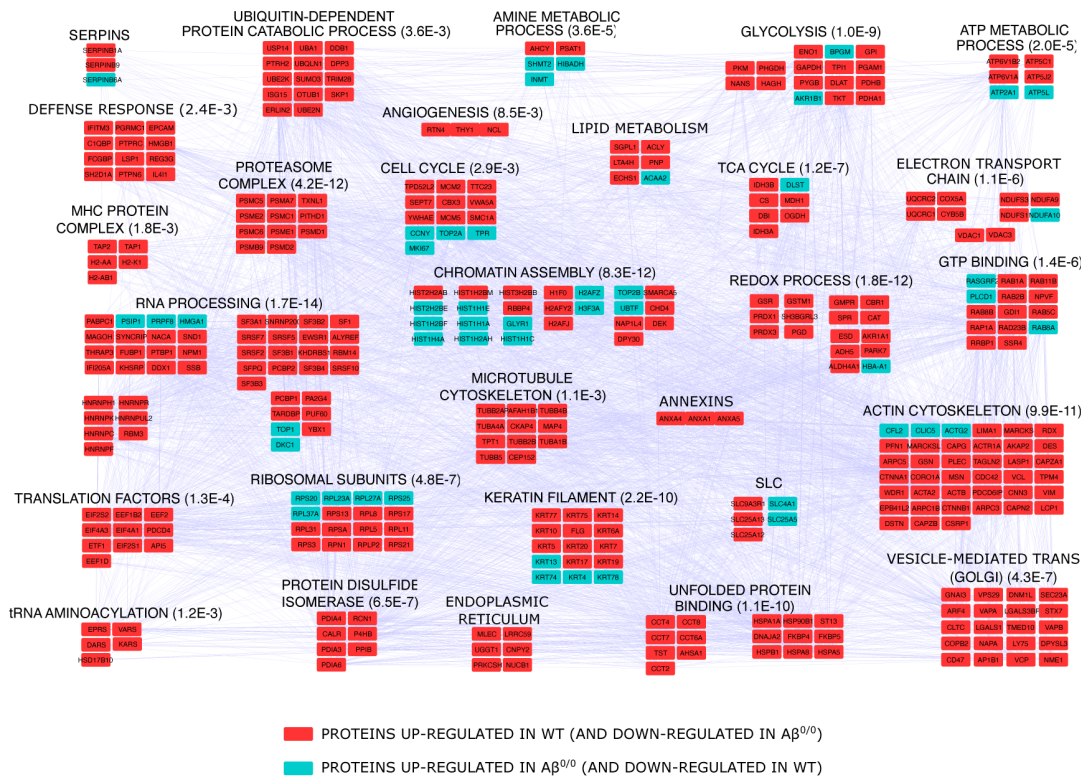

Supplement: Supplementary file 5 [file Image_4.pdf]

# Suppl. Figure 5

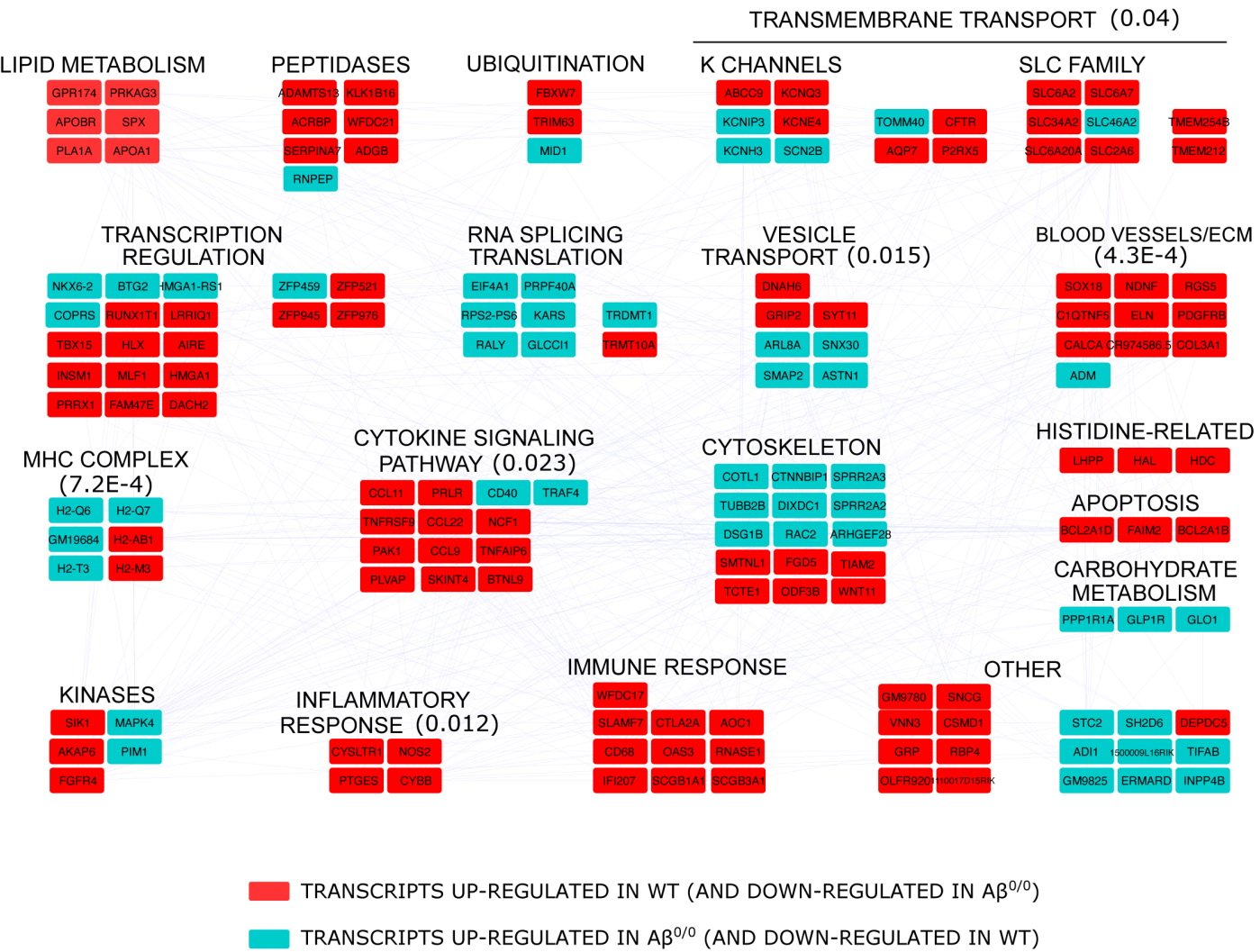

Supplement: Supplementary file 6 [file Image_5.pdf]

Suppl. Figure 6

A

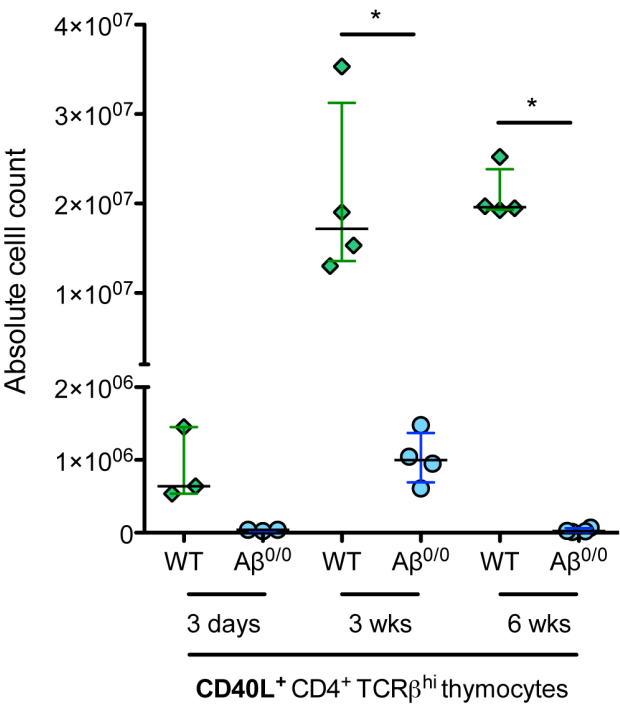

B

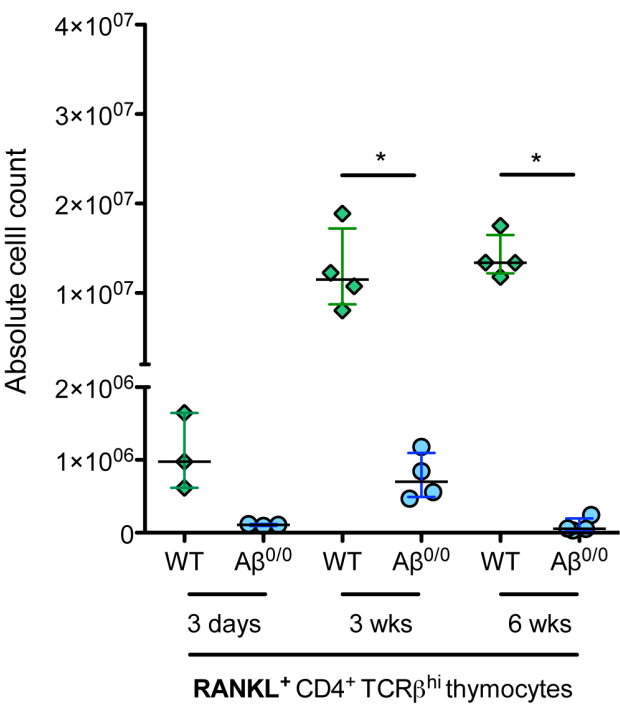

Supplement: Supplementary file 7 [file Image_6.pdf]

# Suppl. Figure 7

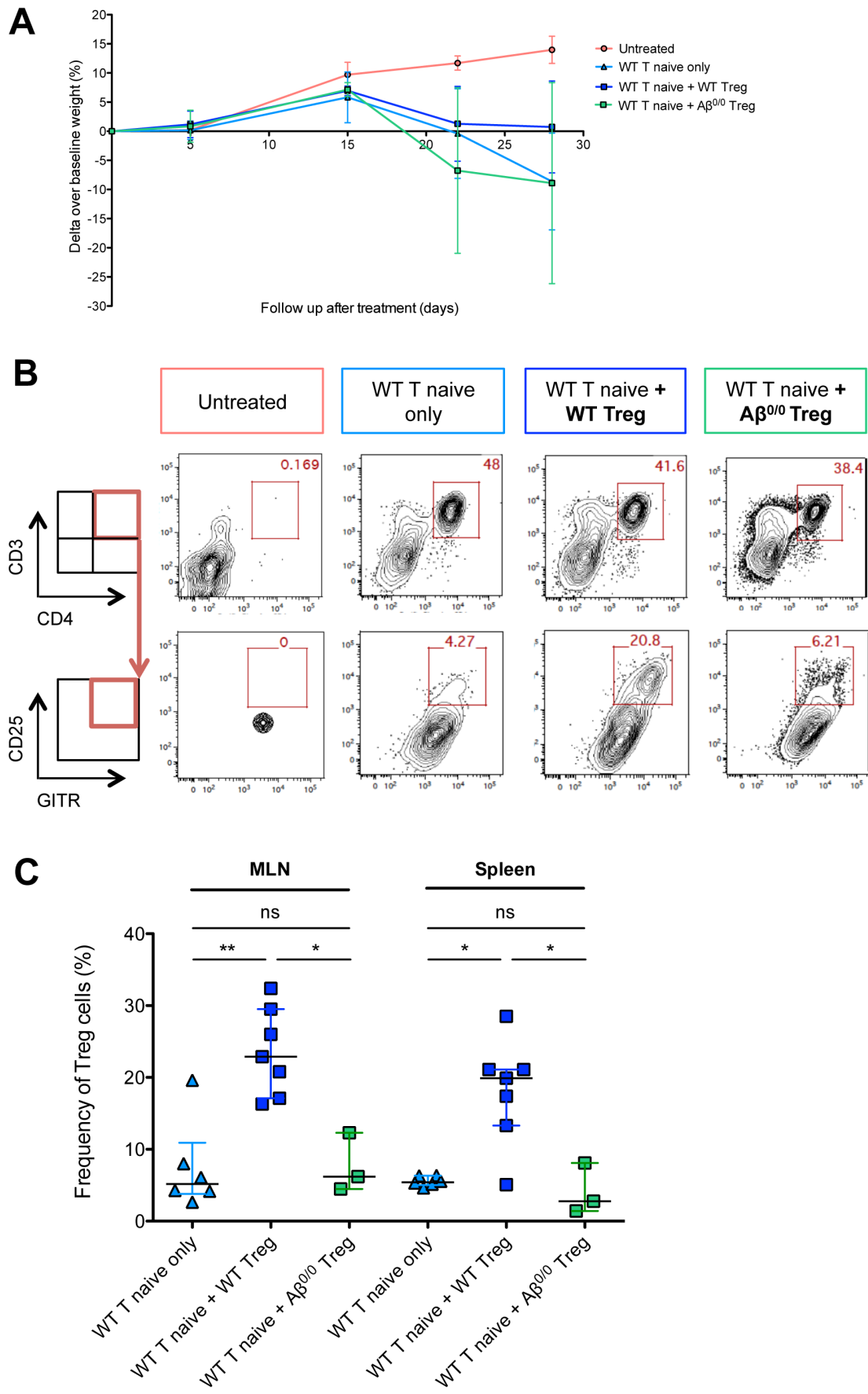

Supplement: Supplementary file 8 [file Image_7.pdf]
